# Quantum Field Approaches to Chemical Systems

**DOI:** 10.1021/jacs.6c02077

**Published:** 2026-09-07

**Authors:** Reza Karimpour, Matteo Gori, Alexandre Tkatchenko

**Affiliations:** Department of Physics and Materials Science, 112680 University of Luxembourg, L-1511 Luxembourg City, Luxembourg

## Abstract

Quantum-matter theory (QMT), based on the Schrödinger or Dirac equations, is firmly established for both intra- and intermolecular interactions. However, there are two key issues with QMT. First, its applicability to large molecular complexes is hindered by the relatively high computational cost of the calculations required to achieve high accuracy. Second, fields are also quantum objects that produce many intriguing effects beyond standard QMT approaches to molecular systems. This review focuses on recent developments in quantum-field theory (QFT) approaches to both covalent and noncovalent interactions for molecules in vacuum and subject to environments such as cavities and solvents. QFT provides a rich playground for novel chemical theories and insights. For example, chemical reactions and van der Waals interactions can be manipulated by cavities, boundaries, and optical excitations; novel interactions emerge when molecules interact with quantized fields; systems with millions of atoms could soon be treated with coarse-grained QFT formalisms; and unexpected scaling laws for atomic and molecular properties can emerge when QFT is applied to sets of chemical systems. This review sets the stage for an exciting QFT-driven path for further development of chemical theory.

## Graphical abstract

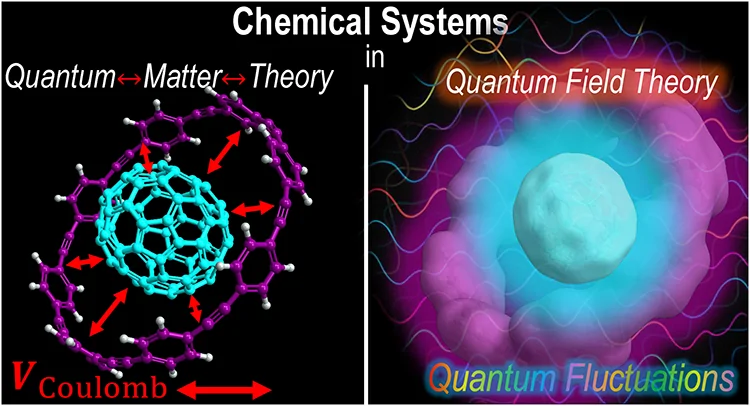

## 1 Introduction

Our ability to model and understand chemistry is intimately tied to the development of quantum mechanics for interacting matter. Quantum-matter theory (QMT), based on the Schrödinger or Dirac equations, is the foundation of current computational methods and conceptual tools for modeling and understanding molecules and materials. The basis for QMT is the Hamiltonian of coupled nuclear and electronic degrees of freedom. Nuclear and electronic charges interact via the instantaneous Coulomb potential, giving rise to many-particle states, including molecular orbitals, molecular vibrations, and molecular plasmons (collective oscillations of the electron density). As many physicists and chemists know, this is an approximation.^1–6^ The quantum vacuum, as the ground state of interacting fluctuating fields of various types, is the fundamental object endowed with its own dynamical degrees of freedom. Quantum field theory (QFT) is the fundamental approach that provides tools for quantizing both matter and fields.^1–3,7^ QFT approaches are not widely used in chemistry because treating the bound states in atoms and molecules is nontrivial in this theory. However, this is slowly changing in the 21st century, with many groups contributing to the brewing of a “chemical QFT revolution”. The importance of QFT techniques is also highlighted by significant advances in quantum information and quantum computing applied to molecular systems.^8–13^

Within QMT, interactions in matter are typically separated into intra- and intermolecular components and are widely studied under different categories, defined by bond types and energy scales.^14–16^ However, the applicability of QMT to large molecular systems is limited by the immense computational cost of the calculations required to achieve the desired accuracy. Furthermore, molecules in cavities or subject to excitations (electric and magnetic fields as well as light) often require *ad hoc* approaches beyond the static Schrödinger equation. Painstaking quantum mechanical (QM) calculations based on coupled-cluster (CC) theory up to triple excitations and/or the quantum Monte Carlo (QMC) method can now reach an unprecedented accuracy of 1 kJ/mol (0.25 kcal/mol or 10 meV) per molecule for systems with a few dozen atoms or molecular crystals.^17–19^ For several important classes of molecular systems, much more efficient semilocal density-functional theory (DFT), including nonlocal many-body dispersion interactions, can achieve predictive accuracy comparable to that of CC or QMC methods when compared with experimental reference data.^20,21^ However, DFT methods are routinely applicable to systems with only 100–1000 atoms.

Despite substantial progress in QMT method development, many fundamental questions remain about the completeness of QMT based on electronic Hamiltonians for large molecular systems,^22–24^ and several puzzles persist even when comparing apparently well-established benchmark QM calculations.^25^ For example, reference CC and QMC calculations are in mutual agreement on the binding energies of molecular complexes containing up to approximately 100 atoms; however, these “benchmark” methods begin to disagree when molecules form supramolecular complexes with a significant contribution from van der Waals (vdW) dispersion interactions to their stability.^25^ For a cycloparaphenylene-ring/buckyball complex containing 132 atoms, the minimal disagreement between the state-of-the-art CC and QMC calculations reaches 31 kJ/mol.^25^ Identifying the reasons behind this unsettling discrepancy requires an improved understanding of electron correlations in many-particle systems over a wide range of spatial scales.

On a more fundamental level, interactions between particles are consequences of their coupling to the quantum fluctuations of vacuum fields. Such fluctuations occur at various scales, but the most relevant to atomic and molecular forces are vacuum electromagnetic field fluctuations.^1–6^ However, QMT treats atoms and molecules as closed quantum-mechanical systems and assumes that electromagnetic fields are fixed classical perturbations.^5^ Although this simplification makes the theory convenient, it also means that the field is not part of the quantum system. Therefore, this semiclassical theory cannot consistently describe essential features, such as vacuum fluctuations and emergent phenomena, including finite speed-of-light effects in dispersion interactions (see Figure 1a), the Lamb shift (Figure 1b), spontaneous photon emission, the well-known Casimir effect between macroscopic ensembles of atoms and molecules (Figure 1b), and the intricate interplay between electronic and photonic degrees of freedom. These phenomena become increasingly pronounced in large systems, strongly coupled molecular ensembles, and in external electromagnetic fields or optical cavity environments.^5,26–32^ The limitations of semiclassical theory become particularly severe when dealing with modern coherent light sources and precision spectroscopy, where the quantized nature of the vacuum field assumes a central role.

**Figure 1:**
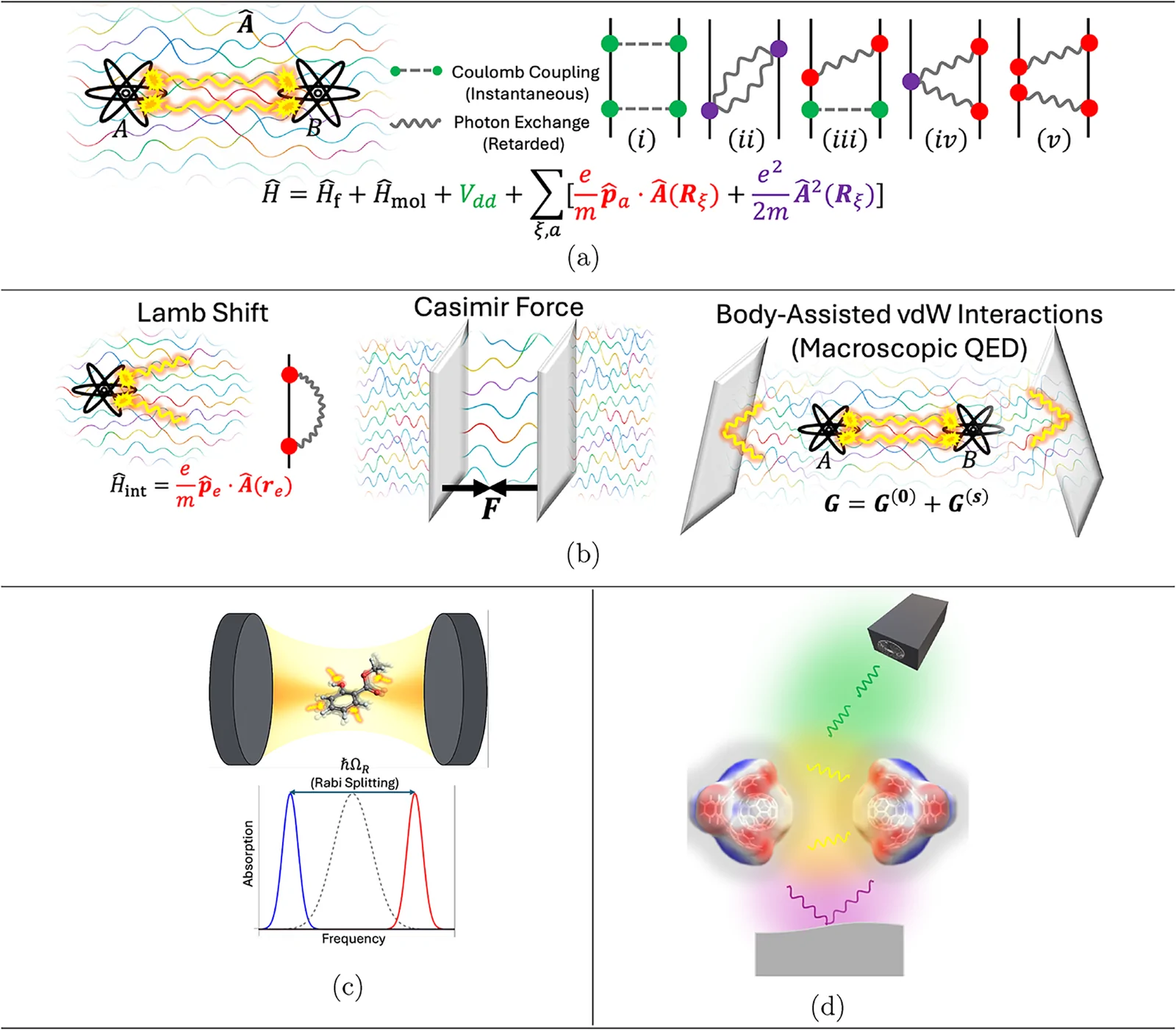
Molecular Interactions from a Quantum Field Theoretical Perspective. (a) Molecular interactions between two polarizable entities and their corresponding QED Feynman diagrams (up to the fourth-order perturbation theory) in the minimal-coupling formalism. Here, *Ĥ*_f_ is the Hamiltonian of the fluctuating electromagnetic field (EMF), *Ĥ*_mol_ denotes the atomic/molecular Hamiltonians, *V*_dd_ is the dipolar Coulomb coupling, and the remaining sum describes atom–field interactions (see Section S3 of the Supporting Information for more details about the Hamiltonian of a system of interacting matter and a quantized electromagnetic field). The diagrams represent processes up to fourth order in perturbation theory. (b) The well-known effects of the vacuum EMF on atoms/molecules, dispersive macroscopic bodies, or the combination of both. The presence of macroscopic bodies and boundaries alters EMF fluctuations, thereby influencing molecular interactions. In macroscopic QED, electric, magnetic, and geometric properties of the boundaries and macroscopic bodies or the bulk materials are encoded into classical Green functions, and the EMF is expressed in terms of that. (c) Inside optical cavities and resonators, strong matter-field couplings are reachable. In such situations, strong coupling between matter and the cavity EMF gives rise to hybrid states that combine properties of molecular and field states, known as polaritons. (d) A quantum-field-theoretical description of matter as a field interacting with other fields (e.g., EMF) allows for the full account of the many-body nature of molecular interactions under the influence of external fields and boundaries and enables the development of computational methods scalable from simple atomic dimers to complex biological macromolecules.

Quantum electrodynamics (QED) overcomes these deficiencies by quantizing both matter and the radiation field within a unified dynamical framework. This methodology provides a rigorous foundation for electron–photon interactions, fully accounting for processes mediated by virtual and real photons. For instance, incorporating QED effects modifies fundamental interatomic dimer potentials, such as altering the vdW dispersion interaction scaling from *R* ^–6^ to *R* ^–7^ due to retardation^33^ at large distances *R*, or to *R* ^–1^ and *R* ^–2^ due to externally induced excitations of the fluctuating electromagnetic field.^26^

Importantly, QED has proven extraordinarily successful in practice. It not only offers conceptual completeness, but also delivers numerical predictions that agree with experiments across a vast range of scales, establishing it as the most accurate physical theory currently available. The QED methodology provides conceptual clarity, enabling a deeper understanding and predictive modeling of quantum correlations beyond traditional particle-like treatments. Recent advances, exemplified by methods such as quantum-electrodynamical density functional theory (QEDFT)^30,34–38^ and coupled quantum Drude oscillator models (cQDO),^39–44^ bridge the gap between highly accurate but computationally demanding techniques (e.g., CC theory and QMC methods) and more efficient but approximate DFT approaches.

The inclusion of quantized electromagnetic fields and their interactions with matter fundamentally modify the electronic structure and response properties of molecules.^45–56^ The significance of such alterations depends on the strength of the photon-matter coupling, which can range from weak to strong and ultrastrong. In the weak-coupling regime, the bare states of atoms and molecules provide a sufficiently accurate description of matter, and the quantized field acts as a perturbation that yields radiative corrections. Well-known examples of phenomena arising from weak atom-field couplings include spontaneous emission, the Lamb shift, Casimir-Polder interactions, and the electron’s anomalous magnetic moment.^1–5^ In contrast, strong and ultrastrong atom-field couplings lead to the formation of photon-matter hybrid states, known as polaritons,^51–53,56^ which give rise to modified potential-energy surfaces and reshaped chemical landscapes. The ability to engineer these surfaces—for example, in optical cavities by tuning properties such as cavity frequency and coupling strength—is the central concept underpinning the emerging field of polariton chemistry.^51,53,56^

An unambiguous signature of strong coupling is Rabi splitting in the absorption spectrum of materials inside cavities as illustrated in (Figure 1c), where the material’s excitation and the cavity photons are tuned into resonance, forming upper and lower polariton states.^57–61^ The formation of polaritons has profound implications, offering novel pathways to influence chemical reactivity^62–67^ and conductivity,^68–71^ shifting conical intersections,^72–74^ or modifying molecular bond lengths.^55,75,76^ Beyond coupling photonic states to molecular electronic or vibrational states, the electromagnetic nature of the fluctuating field also enables matter-field interactions via the magnetic degrees of freedom of these systems. Coupling the quantized field to molecular spins, which are the engines of their magnetic properties, alters spin interactions (e.g., via field-mediated spin–orbit coupling) and effectively retunes the molecules’ magnetic responses, thereby directly influencing their aromaticity.^53,54,77^ A natural framework accounting for such interactions is relativistic quantum electrodynamics, in which spin is inherently incorporated into the quantum-mechanical description of matter through the Dirac equation or its multiple-Fermion extensions.^78^ Another advantage of employing relativistic QED is its ability to achieve precision in describing atomic and molecular systems at the level of fundamental constants, where relativistic and radiative effects become inseparable and measurable in the domain of precision spectroscopy.^79–82^

In addition to external electromagnetic fields, collective interactions among neighboring atoms and molecules strongly influence the molecular response properties. As systems grow in size, many-body interactions–particularly van der Waals dispersion forces–modify the local electronic environment and can significantly enhance molecular polarizabilities. This enhancement is a genuine many-body effect: in one-dimensional carbyne chains, for example, the polarizability of each carbon atom increases by a factor of 50 along the extended structure due to cooperative dispersion interactions.^83,84^ Similar trends are observed in other extended materials, such as graphene, where atoms near the interior of a sheet exhibit larger in-plane polarizabilities than those at the edges.^83^ Consequently, atoms or molecules embedded in large, correlated matter systems interact more strongly with the fluctuating electromagnetic field than their isolated counterparts, thereby amplifying QED effects and radiative corrections. Any realistic treatment of light–matter coupling in extended systems must therefore incorporate these many-body enhancements in the matter subsystem.

In quantum chemistry, approaches that combine density-functional theory with many-body dispersion (MBD) methods^39,42,85,86^ successfully capture these cooperative effects for large molecular assemblies. However, when the electromagnetic field itself is quantized, such techniques no longer apply directly. From a QFT standpoint, matter is naturally described as a field whose excitations constitute the molecular structure, so many-body correlations arise intrinsically within the formalism. A comprehensive description of molecules interacting with quantized electromagnetic fields–whether in cavity vacuum conditions or under external driving–thus becomes a problem of interacting quantum fields (see Figure 1d for an illustration of such a chemical QFT end goal). The advantage of this viewpoint is that the theoretical framework remains unified and scalable, regardless of whether one studies a small organic molecule or a complex macromolecular environment. As experimental control of large molecular systems with confined or structured light continues to advance, the need for a QFT-grounded, many-body–consistent description of molecular systems becomes increasingly apparent.

Before proceeding, we define our terminology to distinguish general theoretical frameworks from specific physical interactions. We use the term “quantum field theory (QFT)” to denote the overarching conceptual framework, as well as the mathematical techniques inspired by field theory, such as second quantization, the representation of matter as continuous fields (e.g., scalar density fields, or vector fields describing matter polarization), and the handling of variable particle numbers in matter and fields. We also use QFT to describe phenomena inherently driven by the quantization of Fermionic matter fields, such as vacuum polarization (fluctuations of electron–positron or other particle–antiparticle pairs), which contributes to the Lamb shift. Conversely, we strictly use the words “quantum electrodynamics (QED)” when referring to the specific subset of QFT that couples matter to the quantized electromagnetic field. Therefore, phenomena driven directly by photon exchange or electromagnetic vacuum fluctuations, such as finite speed-of-light retardation, polaritonic chemistry in cavities, and spontaneous emission, are explicitly termed QED effects. To provide a practical roadmap for these physical boundaries, (Figure 2) presents a decision-tree guide that illustrates when standard QMT is sufficient and when specific QFT or QED frameworks become essential.

**Figure 2:**
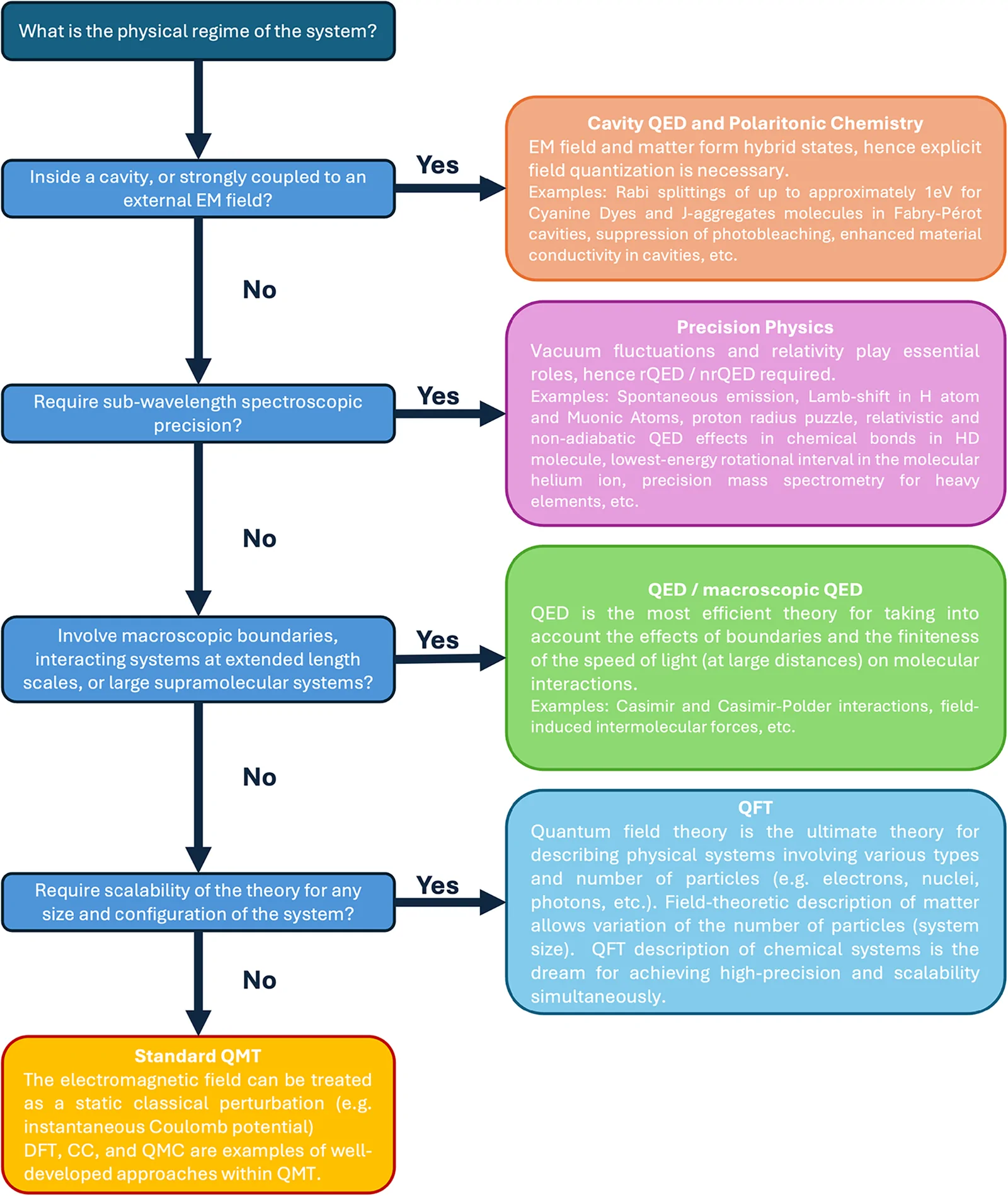
A practical guide for navigating the boundaries between Quantum Matter Theory (QMT) and Quantum Field Theoretical approaches in chemical systems. The flowchart illustrates that standard quantum-chemical methods (e.g., DFT, CC, QMC) remain the highly capable workhorses for standard molecular calculations. However, a transition to specific QED or overarching QFT frameworks is required when the system involves strong external-field coupling (polaritonic chemistry), extreme subwavelength precision (e.g., the Lamb shift), scaling of system size (retardation), or the need for ultimate scalability across varying particle numbers.

In the following, we first summarize the prominent experimental evidence demonstrating the impact of the quantum vacuum on chemical systems. We then present a perspective on QED and QFT methodologies in quantum chemistry. We begin by discussing the conventional quantum-chemistry approach to describing the electronic structure of molecular systems. Subsequently, we explore the advantages of employing field-theoretical methods, such as second quantization and the introduction of particle density (as a scalar field) and polarization tensor fields, in quantum chemistry. We then delve into the description of atoms and molecules in molecular QED, highlighting how QED effects influence noncovalent and covalent interactions (e.g., rotational and vibrational properties of bonds). Finally, we emphasize the importance of QED effects in precision experiments and the key considerations for developing predictive theories for both strong and weak QED effects.

## 2 Concise Summary of Experimental Evidence for Quantum Field Effects in Chemical Systems

From a historical perspective, the most remarkable evidence that a comprehensive theory was required beyond QMT (Schrödinger or Dirac equation for matter) emerged with the observation of splitting in the 2*S*_1/2_–2*P*_1/2_ energy levels of the hydrogen atom.^2,4,45–50^ The QMT, even when employing the Dirac equation, predicts degenerate 2*S*_1/2_ and 2*P*_1/2_ levels for hydrogen.^87^ Lamb and Retherford, using microwave spectroscopy,^45^ measured a small energy difference between these states, with the 2*S*_1/2_ state having a slightly higher energy than the 2*P*_1/2_ state.^46–48^ The inability of the QMT to account for the Lamb shift arises from its neglect of the interaction between the bound electron and the fluctuating electromagnetic field (EMF). One interpretation of the Lamb shift attributes it to the atom’s coupling to the quantized EMF fluctuations, as illustrated in (Figure 1b), which induce variations in the electron’s position and thereby modify the potential it experiences.^2^ Experimental measurements demonstrated that these fluctuations produce energy shifts that are most pronounced in S states, splitting degenerate levels, with the largest observed shift of 1057.8298 MHz for the 2*S*_1/2_–2*P*_1/2_ transition (roughly 10^–4^ kcal/mol). Although this shift appears insignificant in a hydrogen atom, it is worth mentioning that a muonic hydrogen exhibits a Lamb shift of approximately 4.8 kcal/mol due to the significantly higher mass of the muon.^88^ The scaling of the Lamb shift for many-body systems beyond atoms remains unknown at present.

Beyond EMF, the uncertainty principle in quantum mechanics permits fluctuations in all other quantum fields, including both bosonic and Fermionic ones. Within quantum field theory, massive Fermionic fields are a natural generalization of the massless electromagnetic field. While photons can be created and annihilated in QED, general field theories also allow for the creation and annihilation of other massive or massless particles, such as Dirac Fermions.^1–3,7^ Early evidence for Fermionic vacuum effects was provided by Heisenberg and Euler, who examined the low-energy effective action of QED in a constant electromagnetic background and demonstrated that virtual electron-positron pairs polarize the vacuum.^2^ Subsequent calculations and measurements of the Lamb shift^46,50^ confirmed that vacuum polarization induces spectral shifts, such as the 2*S*_1/2_–2*P*_1/2_ splitting of approximately 27 MHz in hydrogen. Although this represents only a fraction of the total Lamb shift in an ordinary hydrogen atom, it is crucial to ensure that theory and experiment match. These effects are particularly significant in muonic atoms, where vacuum polarization dominates, leading to a substantial increase in the vacuum-polarization contribution to the Lamb shift. For example, in muonic helium, vacuum polarization accounts for approximately 90% of the Lamb shift.^89^

Another notable early achievement of QED was its explanation of spontaneous emission. Semiclassical theory suggests that an atom not subjected to an external electromagnetic field does not radiate. Nevertheless, it is well established that a free atom in an excited state will eventually decay to its ground state by emitting a photon. In QED, where the vacuum is characterized by a fluctuating electromagnetic field, this emission is not entirely spontaneous, but is instead stimulated by the vacuum field.^2,4^ The recognition that spontaneous emission can be significantly enhanced or suppressed by the surrounding environment, as proposed by Purcell and termed the Purcell effect, inspired the concept of engineering the vacuum to control matter’s behavior. This insight ultimately led to the development of Cavity-QED,^90^ a field that has become prominent among physicists and chemists for investigating both weak and strong matter-field interactions.

Beyond isolated atoms, precision spectroscopy of small molecules provides a rigorous testbed for QED. Recent measurements of the hydrogen molecule and molecular helium ion (^4^He_2_^+^) have reached unprecedented accuracy, challenging theoretical models. For example, the lowest-energy rotational interval of ^4^He_2_^+^ has been measured as 70.937589 cm^–1^, establishing a benchmark for three-body QED calculations.^79,91^ Discrepancies between theoretical predictions and experimental results in these systems often indicate the need for higher-order QED corrections. In the HD molecule, precision measurements have revealed deviations of 1.4σ to 1.9σ relative to current theoretical values, underscoring the complexity of nonadiabatic and relativistic QED effects in chemical bonds.^92^ Furthermore, the *proton radius puzzle*, a 4% discrepancy in the proton charge radius measured via electronic versus muonic hydrogen spectroscopy, highlights the sensitivity of chemical systems to vacuum field interactions.^82^ These findings confirm that even seemingly simple molecular properties are fundamentally connected to fluctuations in the underlying quantum fields.^79^

In complex chemical systems (more intricate than small molecules or single atoms) confined within cavities, as shown in (Figure 1c), strong coupling between molecules and quantized fields has provided compelling experimental evidence that QED effects alter macroscopic chemical properties. The most direct indicator of strong coupling is the Rabi splitting of the molecular absorption spectrum into upper and lower polariton branches (*P*_+_ and *P*_–_), separated by the Rabi frequency ℏΩ_*R*_. A prominent example of Rabi splitting was observed with cyanine dye molecules and J-aggregates (TDBC) in Fabry–Pérot cavities. In these systems, substantial Rabi splittings of up to approximately 1 eV, or about 0.25 of the resonance frequency, have been measured at room temperature^51,56,93^ (see Figure 5b). These significant Rabi splittings, resulting from strong coupling of molecular electronic states to a cavity’s vacuum field, have been shown to influence photochemical reactivity. For example, the photoisomerization reaction between spiropyran and mecrocyanine was modified by resonant coupling to a Fabry-Pérot cavity, resulting in a Rabi splitting of ℏΩ_*R*_ = 700 meV, which could create a polaritonic barrier that effectively inhibits the reaction rate.^52,62^ Other studies have used plasmonic nanoantennas to generate a strong cavity field that couples to dye molecules, inducing large Rabi splitting that allows excited-state populations to decay faster than they would undergo triplet-state reactions, thereby suppressing the photobleaching of organic chromophores.^52,94^

In addition to electronic transitions, strong coupling of molecules to cavity vacuum fields can also occur via their vibrational degrees of freedom, a phenomenon termed Vibrational Strong Coupling (VSC).^51,52^ By tuning a cavity to resonate with specific bond vibrations, VSC hybridizes molecular vibrational modes with the vacuum field, fundamentally restructuring the ground-state potential energy surface and thereby modifying chemical kinetics and product selectivity without external optical pumping. A fundamental example of this “chemistry in the dark” is the deprotection of 1-phenyl-2-trimethylsilylacetylene (PTA), where resonant coupling to its Si–C stretching mode at 860 cm^–1^ produced a Rabi splitting of 98 cm^–1^ and reduced the reaction rate by a factor of 5.5.^51,52^ Such modifications, which may also include rate enhancement through solvent coupling or the realization of mode-selective chemistry,^52^ are typically enabled by collective coupling that scales with √*N* for *N* molecules.^52,95^ These effects are frequently attributed to *dynamical caging* by the cavity field and have recently been extended to the relativistic regime to control formally forbidden singlet–triplet transitions in heavy atoms such as mercury, providing a noninvasive approach to manipulate intersystem crossing and phosphorescence.^38,78^

It is important to stress that while the aforementioned experimental evidence focuses on specific atomic or molecular benchmarks, QED effects are fundamental and universal; they apply to all atoms, molecules, and materials characterized by many interacting electrons and nuclei. However, the precise scaling of QED phenomena for systems of increasing size remains an open question. In many-body systems, simple dipole-coupling models often fail to capture the nonadditive nature of the interaction, much as the vdW dispersion energy deviates from the standard pairwise interatomic *R* ^–6^ power law in extended nanostructures.^83^ From a QED perspective, rough scaling arguments based on the ***p* ^**· ***A* ^** interaction term (the coupling between momenta in matter and the vector potential of the EMF) suggest that the coupling strength is mediated by the square of the transition dipole moment (|**μ**|^2^). Hence, in large polarizable systems, the delocalization of electrons and the emergence of collective modes can lead to a significant enhancement of this effective coupling, potentially making QED effects far more dominant than in isolated atoms. Identifying and extracting these contributions from experimental measurements remains a formidable challenge, as we currently lack a practical many-body theory capable of disentangling pure vacuum effects from intricate interparticle correlations that are omnipresent in chemical systems.

## 3 Molecules and Fields

The standard formulation of quantum chemistry models electrons as point-like quantum particles and nuclei as classical charges, interacting via the electrostatic Coulomb potential. While wave function-based methods built on this picture have been highly successful, the particle-oriented perspective exhibits intrinsic limitations, particularly in its ability to provide a unified and scalable framework spanning systems from small molecules to large biomolecular complexes in realistic environments. Subsection 3.1 reviews the strengths and limitations of particle-based descriptions of electronic degrees of freedom. Subsection 3.2 introduces a field-theoretical formulation in which both matter and interactions are described by the Schrödinger field for many-body Fermionic systems and the quantized electromagnetic field. Finally, subsection 3.3 presents the conceptual foundations of a more fundamental and unifying framework for nonrelativistic quantum electrodynamics (meaning that matter degrees of freedom are treated non relativistically) in which electronic matter is described by fields and interactions are mediated by fully quantum dynamical degrees of freedom of the electromagnetic field.

### 3.1 Advantages and Limitations of Particle-Based Matter Hamiltonians

The quantum-mechanical description of molecules and materials is commonly based on a particle-based picture in which electrons and atomic nuclei interact via the instantaneous electrostatic Coulomb potential. The significant mass disparity between nuclei and electrons leads to the usual separation of mass scales, making the clamped-nuclei approximation highly effective. Given this approach, nuclei—even when identical—are treated as fixed, distinguishable classical point charges, an assumption typically justified within the Born–Oppenheimer approximation.

In the first-quantized representation, the positions and momenta {***r***, ***p***} of each particle are promoted to Hermitian operators {***r* ^**, ***p* ^**} acting on the Hilbert space of square-integrable functions over real space. These operators satisfy the canonical commutation relations [*r̂*_*k*,*x*_, *p̂*_*k*′,*x*′_] = *i*ℏδ_*kk*′_δ_*xx*′_ *Î* and [*r̂*_*k*,*x*_, *r̂*_*k*′,*x*′_] = [ *p̂*_*k*,*x*_, *p̂*_*k*′,*x*′_] = 0 where *k*, *k*′ label particles and *x*, *x*′ label Cartesian coordinates. Neglecting the trivial Coulomb repulsion between nuclei, the molecular Hamiltonian takes the form

1 $${Ĥ}_{\mathrm{el}‐\mathrm{Coul}}=\sum_{i=1}^{N_{e}}\underset{{\mathrm{K̂}}_{\mathrm{el}}\;\left[{\mathrm{p̂}}_{i}\;\right]}{\underset{︸}{\frac{∥{\mathrm{p̂}}_{i}\;{∥}^{2}\;}{2m_{e}\;}}}+\frac{1}{2}\sum_{\begin{array}{l} i,j=1\\ i\neq j \end{array}}^{N_{e}}\underset{{Û}_{\mathrm{el}-\mathrm{el}}^{\mathrm{coul}}\;\left[{\mathrm{r̂}}_{i}\;,{\mathrm{r̂}}_{j}\;\right]}{\underset{︸}{\frac{e^{2}\;}{4\pi \varepsilon_{0}\;∥{\mathrm{r̂}}_{i}\;-{\mathrm{r̂}}_{j}\;∥}}}-\sum_{i=1}^{N_{e}}\sum_{A=1}^{N_{n}}\underset{{Û}_{n‐\mathrm{el}}^{\mathrm{coul}}\;\left[{\mathrm{r̂}}_{i}\;,R_{A}\;\right]}{\underset{︸}{\frac{Z_{A}\;e^{2}\;}{4\pi \varepsilon_{0}\;∥{\mathrm{r̂}}_{i}\;-R_{A}\;∥}}}$$

Here, *K̂*_el_ denotes the single-particle kinetic energy operator, *Û*_el–el_^coul^ the electron–electron Coulomb interaction, and *Û*_n–el_^coul^ the electron–nuclear interaction potential, where $R_{A}\in R^{3}$ and *Z*_*A*_ denotes the position and atomic number of the *A*-th nucleus, respectively. Equivalent expressions for the nucleus–electron and electron–electron interactions are

2 $${Û}_{n‐\mathrm{el}}^{\mathrm{coul}}=\iint {\mathrm{\rho ̂}}_{\mathrm{el}}\left(r\right)\rho_{n}\left(r^{\prime }\right)V_{\mathrm{Coul}}\left(r,r^{\prime }\right)d^{3}rd^{3}r^{\prime }$$

3 $${Û}_{\mathrm{el}-\mathrm{el}}^{\mathrm{coul}}=\frac{1}{2}\iint :{\mathrm{\rho ̂}}_{\mathrm{el}}\left(r\right){\mathrm{\rho ̂}}_{\mathrm{el}}\left(r^{\prime }\right):V_{\mathrm{Coul}}\left(r,r^{\prime }\right)d^{3}rd^{3}r^{\prime }$$

where *V*_Coul_(***r***, ***r*** ′) = (4πε_0_∥ ***r*** – ***r*** ′∥)^−1^ is the kernel modeling electrostatic Coulomb interactions, ρ̂_el_(***r***) = −*e*∑_*a*=1_^*N*_*e*_^δ^3^(***r* ^**_*a*_ – ***r***) is the electronic density operator, and ρ_n_(***r***) = *e*∑_*A*=1_^*N*_*n*_^ *Z*_*A*_δ^3^(***R***_*A*_ – ***r***) is the nuclear density. Note that self-interaction terms are unphysical and must be removed via regularization of the electronic density product, i.e.,: ρ̂_el_(***r***) ρ̂_el_(***r*** ′) ≔ ρ̂_el_(***r***)­ρ̂_el_(***r*** ′) – (−*e*) ρ̂_el_(***r***)­δ^3^(***r*** – ***r*** ′). It should be noted that all Coulomb interactions in the system can be rewritten in terms of the interaction between the electronic charge density field ρ̂_el_(***r***) = −*e*∑_*a* = 1_^*N*_*e*_^δ^3^(***r* ^**_*a*_ – ***r***) and the electrostatic potential *V̂*(***r***, *t*)­

4 $${Û}^{\mathrm{coul}}={Û}_{n‐\mathrm{el}}^{\mathrm{coul}}+{Û}_{\mathrm{el}-\mathrm{el}}^{\mathrm{coul}}=\frac{1}{2}\int {\mathrm{\rho ̂}}_{\mathrm{Tot}}\left(r\right)\mathrm{V̂}\left(r\right)d^{3}r-\frac{1}{2}\iint \rho_{n}\left(r\right)\rho_{n}\left(r^{\prime }\right)V_{\mathrm{Coul}}\left(r,r^{\prime }\right)d^{3}rd^{3}r^{\prime }$$

where *V̂*[ ρ̂_Tot_(***r***) ]­is a functional of the total charge density operator ρ̂_Tot_(***r***) = ρ̂_el_(***r***) + ρ_n_(***r***) that satisfies the Poisson equations, i.e.

5 $$\nabla^{2}\mathrm{V̂}\left(r\right)=-\frac{{\mathrm{\rho ̂}}_{\mathrm{Tot}}\left(r\right)}{\varepsilon_{0}}\Rightarrow \mathrm{V̂}\left(r\right)=\int V_{\mathrm{Coul}}\left(r,r^{\prime }\right){\mathrm{\rho ̂}}_{\mathrm{Tot}}\left(r^{\prime }\right)d^{3}r^{\prime }$$

Such a formulation of the Coulomb Hamiltonian suggests that at a more fundamental level the interactions between matter degrees of freedom should be described as interactions between *fields*, e.g., the charge density field and the EMF.

Electrons are spin-1/2 Fermions, and the associated electronic wave function ψ­({***r***_*a*_, *s*_*a*_}_*a*=1,...,*N*_*e*__) is defined as the projection of the quantum state onto position–spin eigenstates. A wide range of seminal computational approaches^96–102^ have been developed to solve the time-independent Schrödinger equation associated with eq 1, which constitutes the foundational equation of quantum chemistry in first quantization.

Recent advances in computational methods and experiments have highlighted limitations of this particle-based description, which can be grouped into two categories: (i) the representation of electronic degrees of freedom, and (ii) the treatment of the Coulomb interaction as a static, nondynamical field.

In first quantization, spin statistics are not encoded in the observable algebra and must instead be imposed by antisymmetrizing the electronic wave function. As a result, the many-electron wave function is defined on a 4N_e_-dimensional configuration space, severely constraining the construction of systematically improvable and scalable ansätze.^103,104^ This leads to exponential scaling in wave function-based methods like Full Configuration Interaction, as well as expressivity limitations in Variational Quantum Monte Carlo.^105^ These challenges are partially alleviated by the growing functional expressivity offered by the neural-network wave function ansätze.^106–108^ Further complications arise when the electron number is not fixed, for instance, when exploring molecular chemical space. In this case, the wave function must then be defined over configuration spaces of varying dimensionality. Therefore, projection procedures are required to evaluate real-space observables, including the electron density, whose topological properties encode essential information about chemical bonding (see ref 109 and references therein). In addition, particle-index-based partitions implicit in first-quantized formulations can lead to ambiguities when applying quantum-information measures to systems of identical Fermions^110^ (see Section 4.1 for further details). Moreover, first-quantization picture is not suited for describing relativistic effects for electrons in atoms and molecules. Such relativistic effects have been shown to be particularly relevant for core properties of heavy atoms. For instance, relativistic effects on the ionization energy and electron affinity of the gold atom are comparable in magnitude to electron correlation effects, as estimated at the coupled- cluster CCSD­(T) level.^111^ A consistent description of these effects, and the development of effective relativistic quantum Hamiltonians, require field-based approaches for matter.^112^ These limitations motivate alternative representations of many-body quantum systems of matter particles in terms of fields defined in real or momentum space.

At the same time, the Hamiltonian in eq 1 is structurally limited by its exclusive reliance on electrostatic Coulomb interactions. Here, the EMF does not appear as an independent dynamical quantum object, and energy/momentum exchange via its quantized excitations is excluded at the Hamiltonian level. These limitations are evident when matter degrees of freedom interact with real EMF excitations such as photons and/or electric and magnetic fields. This occurs, for example, with energy transfer in photosynthetic molecular complexes.^4,113–117^ However, EMF excitations are also relevant even without external photons. Intrinsic quantum EMF excitations are carried by “virtual” photons, which can affect both intramolecular and intermolecular interactions.^4,5^ Examples include large-scale charge displacements over long distances^84,118^ in supramolecules or biomolecular complexes, or when EMF–matter coupling is enhanced by specific setups (e.g., molecules in optical cavities),^31,52,119^ or when high computational accuracy is required to predict electronic, optical, or vibrational molecular spectra.^79^ Moreover, leading-order quantum electrodynamic effects for relativistic bound electrons—most notably the Lamb shift—have been conjectured, on the basis of rough estimates, to contribute on the order of ∼1 kcal/mol to the energetics of small molecules containing heavy elements.^120^ The same effects have been shown to produce measurable corrections to predicted single and double ionization energies of 1s and 2s orbitals for elements from the third row of the periodic table.^121^ Taken together, these considerations motivate a reformulation in which both matter and electromagnetic degrees of freedom are described within a QFT framework. In the next section, we introduce the formalism of quantum fields, with particular emphasis on the Hamiltonian formulation of the electromagnetic field; the physical consequences and applications of this extended description are discussed in Sections 4 and 5.

### 3.2 Quantum Description of Matter and the Electromagnetic Field

Although often associated with high-energy or condensed-matter physics, QFT provides a universal language for describing a wide range of many-body quantum systems. It naturally encodes identical particles in 3D space and treats fields (like electromagnetism) as dynamical quantum entities. Crucially, this framework extends to quantum chemistry, offering new insights by unifying many-body effects and correlations under a unified formalism.^122–124^

Conceptually, the particle-based first-quantized formalism is analogous to a Lagrangian description in fluid mechanics, keeping track of individual constituents explicitly, whereas QFT plays a role analogous to an Eulerian description, formulated in terms of (density) fields defined over space.

In a field-theoretic formulation, quantum degrees of freedom are described by fields defined over space. Depending on their nature, such fields may transform as scalars, vectors, or spinors, and their dynamics is governed by partial differential equations in real space.^125–128^ For practical purposes, a basis set of square-integrable field modes {**ϕ**_*k*_(***r***)}_*k*=1,_..._+∞_ is introduced, typically chosen as eigenfunctions of a linear operator describing the corresponding free field, allowing a generic field expansion, i.e., **Φ**(***r***,*t*) = ∑_*k*_ *c*_*k*_(*t*)**ϕ**_*k*_(***r***). Quantization is achieved by promoting the classical field amplitudes *c*_*k*_ to operators acting on a Fock space, introducing annihilation and creation operators {*ĉ*_*k*_, *ĉ*_*k*_^†^}_*k*_ and a vacuum state |0⟩ defined by *ĉ*_*k*_|0⟩ = 0. Acting on the vacuum, *ĉ*_*k*_^†^ creates a single quantum of excitation in mode *k*. The states of the field are then represented in the occupation-number basis |*n*_1_, *n*_2_, ..., *n*_*k*_, ...⟩, defined as eigenstates of the number operators *n̂*_*k*_ = *ĉ*_*k*_^†^ *ĉ*_*k*_. In QFT, the Heisenberg picture is often preferred: fields are treated as dynamical variables, while asymptotic quantum states (in Fock space) are time-independent. Although the interaction picture is commonly used for practical computations in chemical applications (e.g., perturbative response and Fermi’s golden rule), care is needed. Unlike quantum mechanics, the vacuum states of free and interacting Hamiltonians are generally unitarily inequivalent in the continuum limit—a consequence of Haag’s theorem.^128,129^ This formal subtlety underlines why QFT requires careful handling of interactions, even in nonrelativistic chemical settings. Spin–statistics are encoded directly in the operator algebra: bosonic fields satisfy canonical commutation relations, while Fermionic fields obey canonical anticommutation relations. This construction is referred to as *second quantization* ^130^ and forms the basis of field-theoretic approaches to quantum many-body systems.

A key structural distinction between QFT and first-quantized quantum mechanics is particularly relevant for quantum-chemical applications across multiple length scales. In first quantization, spatial coordinates are promoted to operators, and no c-number variables directly represent real-space configurations. In QFT, by contrast, field amplitudes are quantized while spatial coordinates remain c-numbers, enabling a direct real-space description of structure formation and collective behavior, even for systems with arbitrary and fluctuating particle numbers.^126^ Correspondingly, field operators are labeled by spatial coordinates or mode indices rather than by particle indices. As a result, observables such as densities and currents admit a direct real-space representation, without the need for projection procedures. Within this formalism, single-particle orbitals emerge as a choice of basis for expanding the field operators, rather than as explicit building blocks of the many-body wave function. The specific field used to represent the matter degrees of freedom depends on the physical regime: relativistic electrons are described by the Dirac field, whereas nonrelativistic electrons are described by the Schrödinger field (a concise summary of the principal conceptual aspects of quantum field theory is provided in Section S1 of the Supporting Information). As this review focuses primarily on a nonrelativistic treatment of matter degrees of freedom, selected applications of nonrelativistic field-based approaches to quantum chemistry are reviewed in Section 4.

This perspective underlies the conceptual and practical advantages of second quantization for systems of identical particles and enables a unified treatment of matter and electromagnetic degrees of freedom.

### 3.3 General Structure of the Non-Relativistic QED Hamiltonian

By adopting the general framework of QFT, the Hamiltonian in eq 1 can be extended to account for interactions between matter and the EMF treated as a quantum dynamical entity.^4,5^ In this formulation, the electromagnetic degrees of freedom are quantized by applying the second quantization procedure to the vector potential ***A*** (***r***, *t*), from which the observable electric and magnetic fields are obtained as ***E*** (***r***, *t*) = −∂_*t*_ ***A*** (***r***, *t*) – ∇*V*(***r***,*t*) and ***B*** (***r***,*t*) = ∇ × ***A*** (***r***,*t*). The redundancy in the description introduced by the vector potential is eliminated by fixing a gauge. For nonrelativistic systems, a common and convenient choice is the Coulomb gauge where ∇· ***A*** (***r***,*t*) = 0. In the Coulomb gauge, it is convenient to expand the vector potential in plane-wave modes with wavenumber ***k*** as

6 $$Â\left(r,t\right)=\sum_{\lambda =1}^{2}\sum_{k}A\left(k\right)\left[\epsilon \left(k,\lambda \right)e^{-\mathrm{ik}\cdot r}{â}_{k,\lambda }\left(t\right)+\epsilon^{*}\left(k,\lambda \right)e^{\mathrm{ik}\cdot r}{â}_{k,\lambda }^{†}\left(t\right)\right]$$

where $A\left(k\right)={\left[\hbar /\left(2\varepsilon_{0}\omega \left(k\right)V\right)\right]}^{1/2}$ is the quantized field amplitude for a mode of frequency ω­(***k***) = *c*| ***k*** | in a finite volume V (usually introduced to regularize the infrared behavior of the electromagnetic field), and **ϵ**(***k***,λ) are the polarization vectors satisfying the transverse condition ***k*** ·**ϵ**(***k***,λ) = 0 and the completeness relation

$$\sum_{\lambda =1}^{2}\epsilon_{i}^{*}\left(k,\lambda \right)\epsilon_{j}\left(k,\lambda \right)=\delta_{\mathrm{ij}}-\frac{k_{i}k_{j}}{∥k{∥}^{2}}$$

This naturally allows one to define the dyadic matrix projector along the transverse directions $\delta_{\mathrm{ij}}^{⊥}\left(r-r^{\prime }\right)={\left(2\pi \right)}^{-3/2}\int \left(\delta_{\mathrm{ij}}-\frac{k_{i}k_{j}}{∥k{∥}^{2}}\right)e^{\mathrm{ik}\cdot \left(r-r\prime \right)}d^{3}k$ which is important for identifying the dynamical degrees of freedom of the electromagnetic field in the Coulomb gauge.

The operators *â*_***k***,λ_^†^ and *â*_***k***,λ_ are the creation and annihilation operators associated with the mode (***k***, λ), acting on the EMF Hilbert’s space $H_{\mathrm{EMF}}$ .

Within this representation, the Hamiltonian of the free electromagnetic field reads

7 $${Ĥ}_{\mathrm{EMF},\mathrm{free}}\left[{â}_{k,\lambda },{â}_{k,\lambda }^{†}\right]=\frac{\varepsilon_{0}}{2}\int \left[∥Ê\left(r,t\right){∥}^{2}+c^{2}{∥\mathrm{B̂}\left(r,t\right)∥}^{2}\right]d^{3}r=\sum_{k,\lambda }\hbar c\left|k\right|\left({\mathrm{n̂}}_{k,\lambda }+\frac{1}{2}\right)$$

where *c* is the speed of light. The form of the EMF–matter interaction depends on: (i) the representation of matter degrees of freedom (first- vs second- quantized), (ii) the relativistic or nonrelativistic treatment of matter, and (iii) the coupling formulation (minimal vs multipolar). In the first-quantized framework, the interaction is introduced via the *minimal-coupling* prescription: the momentum operator of each charged particle is replaced as ***p* ^**_*a*_ → ***p* ^**_*a*_ – *q* ***A* ^**(***r* ^**_*a*_), where *q* is the particle’s charge, in the Hamiltonian of eq 1. In this expression, the vector potential is evaluated at the particle position operator. An equivalent formulation that preserves an explicit field-theoretic representation can be obtained by expressing the coupling in terms of the electronic current density operator, ***J* ^**_el_(***r***) = −(e/*m*_*e*_)∑_*a*=1_^*N*_*e*_^ ***p* ^**_*a*_δ^3^(***r* ^**_*a*_ – ***r***), together with the electronic charge density operator ρ̂_el_(***r***). The term in minimal-coupling Hamiltonian describing the interaction between electrons and the radiative EMF can then be written as

8 $${Ĥ}_{\mathrm{el}‐\mathrm{EMF}}^{\min}\left[{\mathrm{r̂}}_{a},{\mathrm{p̂}}_{a},{â}_{k,\lambda },{â}_{k,\lambda }^{†}\right]=\sum_{a=1}^{N_{e}}\frac{e}{m_{e}}Â\left({\mathrm{r̂}}_{a}\right)\cdot {\mathrm{p̂}}_{a}+\sum_{a=1}^{N_{e}}\frac{e^{2}}{2m_{e}}{∥Â\left({\mathrm{r̂}}_{a}\right)∥}^{2}=\int \left[{Ĵ}_{\mathrm{el}}\left(r\right)\cdot Â\left(r\right)-\frac{e}{2m_{e}}{\mathrm{\rho ̂}}_{\mathrm{el}}\left(r\right)∥Â\left(r\right){∥}^{2}\right]d^{3}r$$

The choice between relativistic and nonrelativistic treatments of electrons in atomic systems hinges on the ratio of the electron’s characteristic velocity *v* in a given spin orbital to the speed of light *c*. Relativistic corrections to the energy scale as (Z*v*_*H*_/*c*)^2^, and *v*_*H*_/*c* denotes the orbital velocity of the electron in the ground state of hydrogen—expressible as *v*_*H*_/*c* = *a*_Bohr_ *E*_Ha_/(ℏ*c*) ≈ 1/137, with *a*_Bohr_ the Bohr radius and *E*_Ha_ the Hartree energy.^131,132^ Relativistic quantum mechanics becomes increasingly important for accurate core-level energies, and spin–orbit effects while full relativistic QED treatments are only required for high-precision spectroscopic corrections. However, for atoms with *Z* ≤ 30, relativistic corrections to total energies are typically at or below the few-percent level, making nonrelativistic descriptions adequate for many purposes. The total nonrelativistic QED (nrQED) Hamiltonian

9 $${Ĥ}^{\min}={Ĥ}_{\mathrm{EMF},\mathrm{free}}\left[{â}_{k,\lambda },{â}_{k,\lambda }^{†}\right]+{Ĥ}_{\mathrm{el}‐\mathrm{Coul}}\left[{\mathrm{r̂}}_{i},{\mathrm{p̂}}_{i}\right]+{Ĥ}_{\mathrm{el}‐\mathrm{EMF}}^{\min}\left[{\mathrm{r̂}}_{i},{\mathrm{p̂}}_{i},{â}_{k,\lambda },{â}_{k,\lambda }^{†}\right]$$

provides a widely applicable and consistent description of EMF–matter interactions, applicable to both extended materials and molecular systems. In its minimal-coupling form, matter degrees of freedom interact locally with the electromagnetic vector potential, making this formulation particularly suitable for systems with delocalized charges and translational invariance.

From the structure of eq 9, the coupling between matter and the electromagnetic field appears to be set by the electric charge *q*, as dictated by the minimal-coupling prescription. However, the effective strength of this interaction can be expressed in terms of a dimensionless parameter, α. In the low-energy regime—where particle–antiparticle creation and annihilation processes are negligible—α reduces to the *fine-structure constant*

10 $$\alpha =\frac{e^{2}}{4\pi \varepsilon_{0}\hbar c}≃\frac{1}{137}$$

The smallness of α underlies the success of perturbative approaches in low-energy quantum electrodynamics involving a limited number of charged particles. By contrast, in systems exhibiting strong electronic correlations—arising from high particle densities or specific geometric arrangements—effective coupling strengths can be significantly enhanced, and nonperturbative matter–EMF effects may emerge, as discussed in Section 5.

For systems composed of spatially localized and distinguishable assemblies of bound charges—such as atoms, molecules, or molecular aggregates—it is often advantageous to adopt an alternative description of the EMF–matter interaction, inspired by the multipolar framework. In this approach, the matter degrees of freedom are described not by individual particle coordinates, but by macroscopic polarization and magnetization fields. This provides a mathematically rigorous formulation of the intuitive idea: that confined charge distributions can be effectively represented through their multipole moments (dipoles, quadrupoles, etc.). This motivates the use of the *multipolar coupling* Hamiltonian, obtained by applying the Power–Zienau–Woolley (PZW) unitary transformation to the combined matter–EMF system.^4,5,133^ The PZW transformation recasts the EMF–matter interaction in terms of collective polarization and magnetization fields associated with spatially localized subsystems. For each localized charge set ξ, the interaction is described by the polarization field operator ***P* ^**_ξ_(***r***,*t*), defined such that ρ̂_ξ_(***r***,*t*) = −∇· ***P* ^**_ξ_(***r***,*t*), together with the magnetization field operator ***M* ^**_ξ_(***r***,*t*) satisfying ∇ × ***M* ^**_ξ_(***r***,*t*) = ∂_*t*_ ***P* ^**_ξ_(***r***,*t*) – ***J* ^**_ξ_(***r***,*t*) and the diamagnetic susceptibility tensor ***O* ^**_ξ_(***r***, ***r*** ′) (see Section S3 of the Supporting Information). The multipolar gauge, introducing a description of the quantum electronic degrees of freedom in terms of molecular polarization field, provides a rigorous and conceptually consistent framework for embedding and developing approaches to intermolecular noncovalent dispersion interactions.^134,135^

It is worth noting that the constitutive relations defining the polarization and magnetization fields do not uniquely fix these fields themselves, but only the associated charge and current densities. As a consequence, the polarization and magnetization fields are defined only up to transformations that leave the physical sources invariant.^133^ Such a gauge freedom reflects the fact that different choices of polarization and magnetization fields can represent the same underlying charge distribution. Formally, it constitutes the mathematical counterpart of the arbitrariness inherent in the choice of reference point (or reduction pole) used in the multipole expansion of an extended electronic charge distribution.

In the multipolar gauge, the nonrelativistic QED Hamiltonian can be written as

11 $${Ĥ}_{\mathrm{nrQED}}^{\mathrm{mult}}={Ĥ}_{\mathrm{EMF},\mathrm{free}}+\sum_{\xi }\left\{\sum_{a_{\xi }\;\in \xi }\left[{\mathrm{K̂}}_{\mathrm{el}}\left[{\mathrm{p̂}}_{a_{\xi }}\right]+{Û}_{\mathrm{el}‐\mathrm{el}}\left[{\mathrm{r̂}}_{a_{\xi }}\right]+\sum_{A_{\xi }\;\in \xi }{Û}_{n‐\mathrm{el}}\left[{\mathrm{r̂}}_{a_{\xi }};{\mathrm{R̂}}_{A_{\xi }}\right]\right]+\frac{1}{2\epsilon_{0}}\int {∥{\mathrm{P̂}}_{\xi }^{⊥}\left(r\right)∥}^{2}d^{3}r-\int \left[\varepsilon_{0}^{-1}{\mathrm{P̂}}_{\xi }^{⊥}\left(r\right)\cdot {\mathrm{D̂}}^{⊥}\left(r\right)+\mathrm{B̂}\left(r\right)\cdot {\mathrm{M̂}}_{\xi }\left(r\right)\right]d^{3}r+\frac{1}{2}\iint \mathrm{B̂}\left(r\right)\cdot {Ô}_{\xi }\left(r,r^{\prime }\right)\cdot \mathrm{B̂}\left(r^{\prime }\right)d^{3}rd^{3}r^{\prime }\right\}$$

where the displacement vector field ***D* ^**(***r***,*t*) = *ε*_0_ ***E* ^**(***r***,*t*) + ***P* ^**(***r***,*t*) has been introduced, and the transverse component $v_{i}^{⊥}\left(r\right)=\sum_{j}\int \delta_{\mathrm{ij}}^{⊥}\left(r-r^{\prime }\right)v_{j}\left(r^{\prime }\right)$ of a a vector field ***v*** (***r*** ′) d^3^ ***r*** ′ (see the Supporting Information for further details). Within this formulation, each localized matter subsystem couples directly only to the EMF, while effective matter–matter interactions arise from field-mediated processes. This structural feature makes the multipolar gauge particularly well suited for describing molecular spectroscopy, polarization phenomena, and EMF–matter interactions in confined or cavity environments.

It is worth emphasizing that, in both the minimal-coupling and multipolar formulations, the matter-EMF coupling can be rewritten just in terms of the *matter density fields* rather than in terms of individual particles. In a first-quantized description, these fields reduce to singular distributions supported at the particle coordinates, i.e., Dirac delta functions. By contrast, within a second-quantized formulation of matter, the electric charge and current densities become operator-valued fields and admit a mode expansion in terms of the underlying matter field operators. The particle-based description is recovered as a particular limiting case of this field-theoretic formulation, corresponding to states with fixed and pointwise localized particle content.

In the following sections, we illustrate the impact of field-inspired methods in quantum chemistry. We show how second quantization provides a natural framework for quantum-information analyses of electronic structure, yielding direct insight into covalent bonding through field-theoretical observables such as the energy–stress tensor. We further review how effective field-based Hamiltonians enable coarse-grained descriptions of electronic density and correlations (Section 4.2), supporting multiscale modeling and the derivation of scaling laws linking microscopic electronic correlations to macroscopic physicochemical properties.

## 4 Quantum Field Description of Atoms and Molecules

This section reviews representative applications of field-oriented descriptions of electronic quantum matter in chemistry. Subsection 4.1 revisits the Schrödinger field formalism—namely, the occupation-number representation of spin orbitals—in quantum chemistry, with applications to the analysis of chemical bonding, including approaches based on quantum information theory. Subsection 4.2 examines effective field-theoretical models of electronic degrees of freedom, with particular emphasis on density functional theory and the second-quantized formulation of many-body dispersion interactions. Finally, Subsection 4.3 discusses how field- oriented formulations of the electronic structure problem enable the derivation of scaling laws for key physicochemical observables, such as molecular polarizability, across a broad range of molecular sizes.

### 4.1 Field-Inspired Approaches to Matter Hamiltonian and Chemical Bonding

Different possible QFT representations of the electronic degrees of freedom have been applied in the context of quantum chemistry, depending on the desired level of accuracy (e.g., whether relativistic effects must be included): 4-component Dirac spinor field,^78^ Schrödinger wave function field, or charge-density field. The Schrödinger field representation of the electronic degrees of freedom is the closest quantum field theoretical approach to the usual wave function methods.^142,143^ The second quantization scheme presented in the previous section can be applied to the Schrödinger field, where the electronic degrees of freedom are expressed in terms of orthonormal spin orbitals, **ϕ**_*s*, η_(***r***) = ϕ_η_(***r***) **v**_*s*_, where {ϕ_η_(***r***) }_η=1,...,+∞_ denotes a chosen complete basis set of square-integrable functions on a domain in real space while **v**_↑_ = (1,0)^*T*^ and **v**_↓_ = (0,1)^*T*^ are the spin vectors. The corresponding (adjoint) field operator is

12 $${\mathrm{\psi ̂}}^{\left(†\right)}\left(r\right)=\sum_{s=↑,↓}{\mathrm{\psi ̂}}_{s}^{\left(†\right)}\left(r\right)=\sum_{\eta \in O^{\mathrm{mol}}\;}\phi_{s,\eta }^{\left(*\right)}\left(r\right){ĉ}_{\eta ,s}^{\left(†\right)}$$

where *ĉ*_η,*s*_^†^ and *ĉ*_η,*s*_ are the Fermionic creation and annihilation operators for the spin orbital labeled by (η,*s*). Acting on Fock space, these operators generate many-electron basis states of the form |*n*_η1_, *s*_1_, ..., *n*_*ηk*_, *s*_*k*_, ...⟩, labeled by the occupation number of each spin orbital. In such a QFT picture, the electric charge and current density distributions read

13 $${\mathrm{\rho ̂}}_{\mathrm{el}}\left(r\right)=-e\sum_{s=↑,↓}{\mathrm{\psi ̂}}_{s}^{†}\left(r\right){\mathrm{\psi ̂}}_{s}\left(r\right)=-e\sum_{s=↑,↓}\sum_{\eta ,\eta^{\prime }\;\in O^{\mathrm{mol}}\;}\phi_{\eta \prime }^{*}\left(r\right)\phi_{\eta }\left(r\right){ĉ}_{\eta \prime ,s}^{†}{ĉ}_{\eta ,s}$$

14 $${Ĵ}_{\mathrm{el}}\left(r\right)=\frac{\mathrm{ie}\hbar }{2m_{e}}\sum_{s=↑,↓}\left[{\mathrm{\psi ̂}}_{s}^{†}\left(r\right)\nabla_{r}{\mathrm{\psi ̂}}_{s}\left(r\right)-\left(\nabla_{r}{\mathrm{\psi ̂}}_{s}^{†}\left(r\right)\right){\mathrm{\psi ̂}}_{s}\left(r\right)\right]=\frac{\mathrm{ie}\hbar }{2m_{e}}\sum_{s=↑,↓}\sum_{\eta ,\eta^{\prime }\;\in O^{\mathrm{mol}}\;}\left[\phi_{\eta \prime }^{*}\left(r\right)\nabla \phi_{\eta }\left(r\right)-\left(\nabla \phi_{\eta \prime }^{*}\left(r\right)\right)\phi_{\eta }\left(r\right)\right]{ĉ}_{\eta \prime ,s}^{†}{ĉ}_{\eta ,s}$$

Such a second-quantized description of the nonrelativistic electronic wave function forms the foundation of modern electronic structure methods, including Hartree–Fock theory and the CC approach,^144,145^ for which second quantization constitutes a fundamental basis. Recently, renewed interest in field-based formulations of CC theory has highlighted the connections to algebraic geometry,^146,147^ offering new perspectives on the structure and solvability of the coupled-cluster equations. Moreover, the second-quantization formulation of the electronic Hamiltonian in quantum chemistry enables the determination of ground-state properties using quantum computational methods.^148–152^ In particular, the Jordan–Wigner transformation provides a mapping from Fermionic modes to qubit operators, thereby allowing the quantum chemistry Hamiltonian to be expressed as a sum of qubit operations.

Recent research highlights the second-quantized framework as a natural, powerful setting for applying quantum information tools to quantum chemistry. Central to this is the concept of *orbital entanglement*:^110,138,153–155^ in identical-particle systems, subsystems are defined not by partitioning particles, but by partitioning field modes—e.g., electronic spin–orbitals. Operators for a single Fermion (e.g., an electron) do not form a valid subsystem in many-body Fermionic systems^110^ from which it follows that first-quantized reduced density matrices (RDMs)—obtained by tracing out electron positions—are conceptually problematic. In contrast, the algebra of creation/annihilation operators for a single orbital defines a proper subsystem, enabling consistent 1- and 2-orbital RDMs. Some care is required in dealing with Fermionic RDMs, as creation and annihilation operators for different orbitals do not commute in general—a feature consistent with the microcausality principle in nonrelativistic settings.^156^ This implies that superselection rules must be applied to the RDM, forbidding superpositions of Fermionic states with different particle-number parity—the so-called parity superselection rule (P-SSR)—or preserving the total number of Fermions—the number superselection rule (N-SSR).^154,155^ Within this framework, quantum information methods yield new insights—e.g., via *orbital* entanglement estimators applied across spin–orbital bases to identify those best describing covalent bonds, bridging molecular orbital and valence bond theories. Atomic orbital bases maximizing orbital entanglement estimators are most suited (see panel (a) of Figure 3) for characterizing covalent bonds. Based on this, a new method assesses active orbital space quality in Complete Active Space Configuration Interaction,^157,158^ yielding results analogous to first-quantized CC via Domain-Based Local Pair Natural Orbital.^159,160^

**Figure 3:**
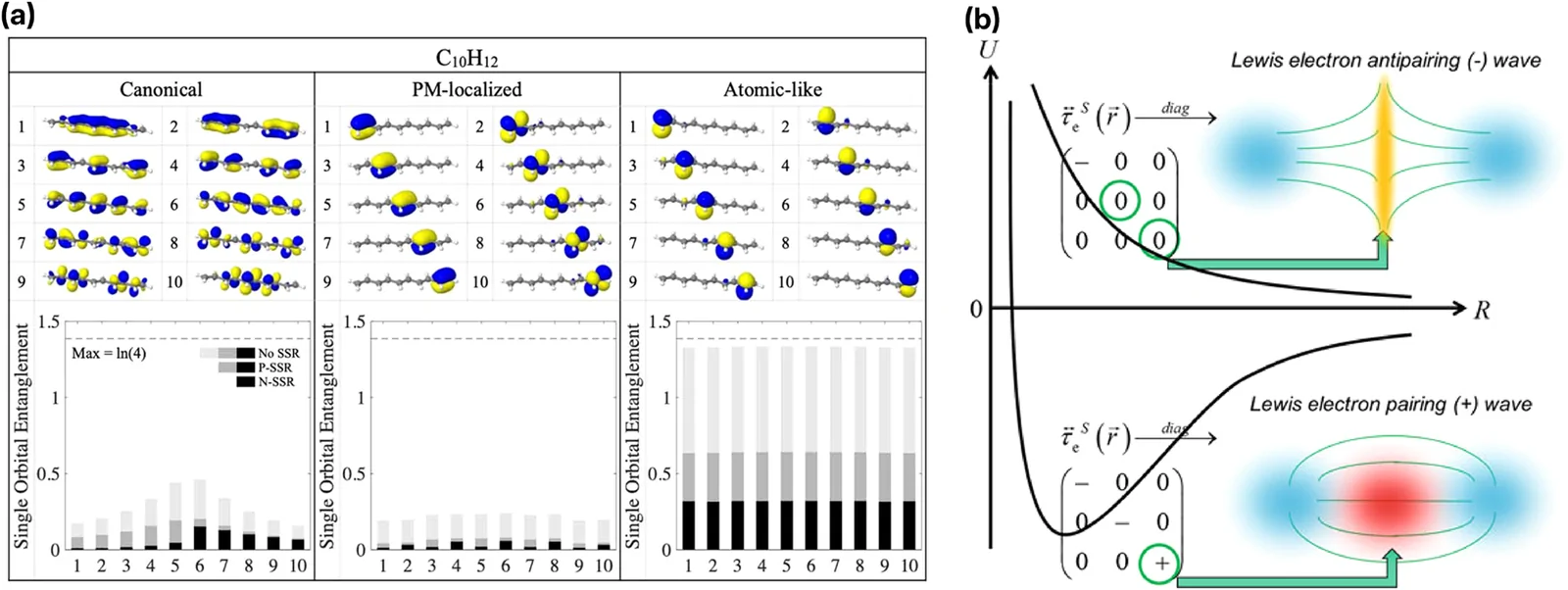
Applications of QFT-based methods to Quantum-information and electronic-stress analyses of molecular bonding. (a) Single-orbital entanglement (quantum relative entropy) in the Complete Active Space calculations correlating 10 electrons in 10 π orbitals for C_10_H_12_. The orbital numbering follows the upper panel (canonical from self-consistent Hartree–Fock/from Pipek-Mezey (PM) localization^136^/atomic by Jacobi-rotation of PM π-orbitals^137^). Colors indicate no Superselection Rules (SSR) (all colors), Parity-SSR (black/dark gray), and Number-SSR (black). Reproduced from ref 138. Available under a CC-BY 4.0 license. Copyright 2022 L. Ding, S. Knecht, Z. Zimboras, and C. Schilling. Published by IOP Publishing Ltd. (b) Bonding/antibonding characterization in H_2_-like dimers via eigenvalues and flow lines of the electronic stress tensor ${\mathrm{\tau ⃡}}_{e}\left(r\right)$ : the 1*sσ* state exhibits tensile stress (>0) at the bond midpoint and a spindle structure in the stress lines, whereas 1sσ*shows compressive stress (<0) and a disrupted stress topology consistent with antibonding character. Reproduced with permission from ref 139. Copyright 2018 John Wiley and Sons.

The field-theoretical approaches for describing electronic quantum states allow one to adapt methods derived from continuous mechanics and quantum fluid mechanics to gain a deeper understanding of chemical bonds. A remarkable example in this sense is the “rigged” QED formalism for describing covalent and hydrogen bonding (see refs 161,162 and references therein for a comprehensive perspective). In this approach—particularly within the *primary* rigged QED framework tailored for quantum chemistry—both electrons and nuclei are treated as quantum Schrödinger fields coupled to the quantized electromagnetic field. Evaluating these fields in the ground state yields classical field representations, from which the real-space energy projection—the system’s rank-2 stress–energy tensor—is derived. Analyzing the flow lines of its principal eigenvector reveals a characteristic spindle-shaped pattern signature of covalent bonding (see panel (b) of Figure 3). Recent extensions have captured subtle features of hydrogen bonding,^163^ underscoring the power of quantum field-based descriptions of matter to uncover the field-theoretic roots of molecular interactions. Field-inspired approaches are effective in describing the real-space embedding of electronic structure properties beyond the local scale of covalent bonds.

Because position in real space functions as a label rather than a quantum variable, field-theoretic formulations inherently span all length scales. When applied to the electronic-structure problem in eq 1, these approaches provide new insight into electronic correlation, particularly in the treatment of long-range, noncovalent interactions.

### 4.2 Non-Covalent Interactions from Effective Field Models for Electronic Density and Polarization

A field-theoretic formulation of electronic degrees of freedom grants direct access to real-space densities of physical observables, enabling spatially resolved characterization of intra- and intermolecular interactions. Central among these is the electronic charge density which underpins most molecular properties. The electron density is both computationally tractable and experimentally measurable—notably via X-ray diffraction and quantum tomography—and its geometric and topological features have been systematically analyzed to decode chemical bonding mechanisms.^109,164^ The centrality of the expectation value of the charge density field ρ_el_(**r**) = ⟨ρ̂_el_(**r**)⟩_GS_ in the ground state is formalized by the Hohenberg–Kohn theorem: a one-to-one mapping exists between the nuclear potential *Û*_n–el_(**r**) and ρ_el_(**r**), derived from the Hamiltonian in eq 1. This implies an energy functional *E*[ρ_el_] = F­[ρ_el_] + *U*_el–*n*_[ρ_el_], where *F*[ρ_el_] = *T*_*s*_[ρ_el_] + *E*_H_[ρ_el_] + *E*_xc_[ρ_el_] is universal—encoding kinetic, Hartree, and exchange–correlation contributions—yet remains unknown in exact form. In Kohn–Sham DFT, the exchange-correlation functional *E*_xc_[ρ_el_], in principle, accounts for all beyond-Hartree correlations. Its form, especially for what concerns long-range component—critical for nonlocal effects—remains the principal modeling challenge, motivating advanced approximations. QFT methods provide a first-principles route to *F*[ρ_el_]: DFT arises naturally as an effective low-energy field theory for the density operator^165,166^ —in the sense of an effective action for the density–enabling perturbative constructions of the exchange-correlation functional *E*_xc_. Nonperturbative approaches, inspired by functional renormalization, have recently yielded 3D universal functionals.^167^ Despite decades of progress in constructing increasingly accurate energy density functionals, no universally applicable functional yet exists that reliably captures the contribution of electronic density correlations to interaction energies—particularly in noncovalent, many-body regimes. A promising strategy consists in defining model integrable Hamiltonians encoding electronic density correlations via physically motivated representations. A prominent example is the Many Body Dispersion (MBD) model.^39,168–170^ In this framework, the electronic response—derived from a chosen density functional approximation (DFA)—is mapped onto a system of quantum Drude oscillators (QDOs), which interact via a dipole–dipole electrostatic potential. The MBD Hamiltonian thus provides a low-energy effective description of long-range electronic correlations, typically manifesting at length scales ≫5 Å, through the collective normal modes of the coupled QDO system. This formalism yields computationally efficient, physically transparent expressions for the MBD energy—a contribution systematically absent in semilocal DFAs—enabling systematic studies of dispersion effects in large molecular complexes and extended materials. Moreover, MBD has been validated as a quantitative proxy for charge density distortion relative to high-accuracy references (e.g., CCSD­(T)), when initialized with DFA-derived polarizabilities.^118^ A second-quantized formulation of MBD, inspired by quantum field theory, was recently introduced.^42^ It maps collective MBD modes—and the ground state of the interacting QDO system—onto atomic QDO displacements and noninteracting eigenstates via a Bogoliubov transformation. This allows one to derive a fragment-resolved decomposition of the MBD energy (see panel (a) of Figure 4), and provides a rigorous basis for applying quantum information tools to quantify correlation pathways in the MBD-induced charge density.^42^

**Figure 4:**
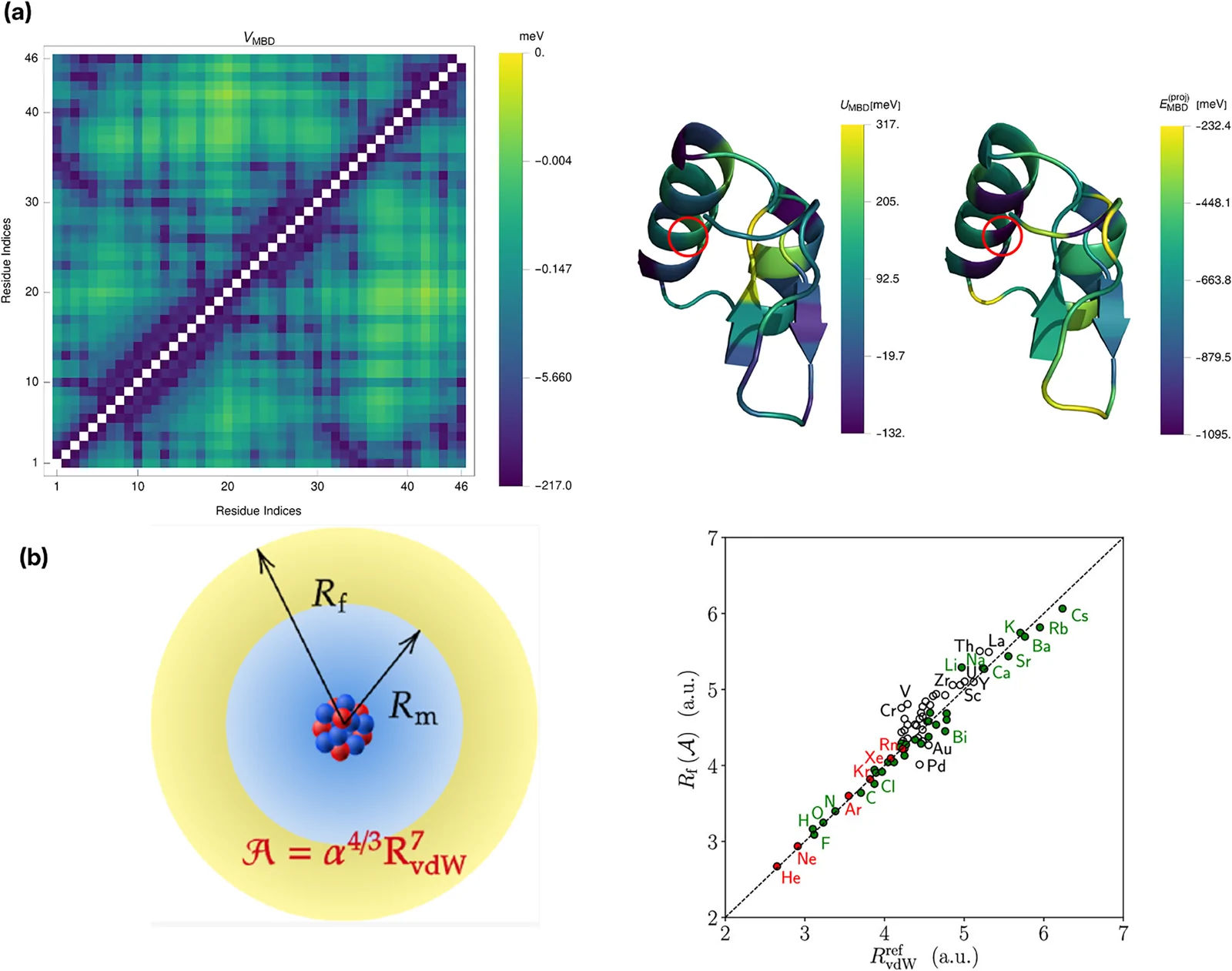
Applications of QFT-based methods to Quantum-information and electronic-stress analyses of molecular bonding. (a) Second Quantized Many-Body (SQ-MBD) decomposition of the Many-Body dispersion energy into intrafragment (*U*_MBD_), interfragment pair (*V*_MBD_), and per-fragment (*E*_MBD_^Frag^) contributions, shown per residue in crambin (meV).^42^ Reproduced from ref 42. Available under a CC-BY 4.0 license. Copyright 2023 M. Gori, P. Kurian, and A. Tkatchenko. Published by Springer Nature. (b) Reference van der Waals radii *R*_vdW_^ref^ ^140^ versus electromagnetic field (EMF)-dressed radii for 72 elements, and corresponding polarizabilities (in atomic units); noble gases (red), transition metals (open symbols), and other elements (green). The plot illustrates the scaling relation between static polarizability A and the EMF-dressed van der Waals radius *R*_f_, with a prefactor involving the fine-structure constant α. Reproduced from ref 141. Available under a CC-BY 4.0 license. Copyright 2021 A. Tkatchenko, D. Fedorov, and M. Gori. Published by American Chemical Society.

These examples indicate that a field-based formulation offers a natural and systematic framework for the construction of effective Hamiltonians describing matter degrees of freedom across different length and energy scales. In this context, a compelling extension of the MBD framework is to introduce an effective Hamiltonian for quantum electronic charge-density fluctuations defined with respect to a reference semiclassical electronic density obtained from DFT. Conceptually, this approach parallels semiclassical approximations in the path-integral formulation of quantum field theory, where the dynamics of fluctuations are treated perturbatively around a saddle-point configuration. This formulation would transcend the QDO paradigm by enabling an optimized and self-consistent determination of the spatial distribution, parametrization, and coupling constants of the effective oscillators, rather than prescribing them through semiempirical models.

Field-inspired approaches provide a natural bridge between atomistic and molecular length scales. The systematic connection across these scales is most effectively captured through the derivation of scaling laws—quantitative relationships that encode how physical properties evolve with system size, number of particles, composition, or geometry.

### 4.3 Scaling Laws for Covalent and Non-Covalent Interactions from Field Theory Perspective

A central limitation of first-quantized quantum mechanics in the study of scaling laws is its reliance on a fixed-particle-number description. When the number of electrons changes across systems—an unavoidable situation when exploring chemical space—scaling relations for quantum-mechanical observables such as energies, multipole moments, or polarizabilities cannot be formulated in a unified manner. By contrast, QFT-inspired approaches operate within an occupation-number representation, naturally embedding Hilbert spaces corresponding to different particle numbers and thereby providing a consistent framework for analyzing trends across families of molecular systems.

This perspective underlies field-theoretic and field-inspired approaches to chemical bonding. A notable example is Alchemical Perturbation Density Functional Theory (APDFT), which exploits functional derivatives of the ground-state DFT energy and electron density with respect to nuclear density to define an *alchemical potential*.^171,172^ This observable quantifies the response of electronic structure to changes in nuclear charge and provides chemically meaningful insight into bonding trends, such as variations in acidity or reactivity. By enabling the exploration of entire families of isoelectronic compounds without explicit enumeration, APDFT illustrates how field-theoretic concepts allow systematic navigation of chemical space.

Field-theoretic methods provide a natural route to derive scaling laws for extensive response properties, due to flexibility of these methods in describing systems with different number of particles. In particular, recent work has shown that the static polarizability A of systems governed by central potentials scales as $A\propto L^{4}/a_{0}$ , where *L* is the characteristic length scale of the ground-state charge density and *a*_0_ the Bohr radius, rather than following a naive volume scaling.^173,174^ This result underpins the theoretical derivation of the empirical relation $R_{\mathrm{vdW}}∼A^{1/7}$ for vdW radii^141^ (see panel (b) of Figure 4). Importantly, the proportionality constant in this scaling law encodes the fine-structure constant α, revealing that vdW radii originate from a quantum electrodynamical dressing of electronic degrees of freedom by low-energy virtual photons.

These examples show that scaling laws for both covalent and noncovalent interactions arise naturally when the electronic structure is formulated within a field-theoretic framework. A central challenge moving forward is the development of fully first-principles, QFT-based approaches capable of deriving scaling relations for a broad range of observables across molecular systems spanning vastly different length scales. A particularly important direction is the extension of scaling laws linking the real-space geometry of the electronic charge density to extensive response properties, such as the static polarizability. These properties govern long-range interactions and collective phenomena in large molecular assemblies, including biomolecular complexes comprising millions of atoms in realistic aqueous environments. Understanding how electronic and nuclear charge distributions in real space determine electronic-structure properties is therefore essential to develop accurate coarse-grained interaction models and to build chemical fragment databases to train machine-learning force fields with quantum-chemical accuracy.^175,176^

The results presented in this section demonstrate the power of the QFT formalism and techniques in providing a unified understanding of quantum chemistry, as described by eq 1, across multiple length scales. However, as systems are scaled to larger and more complex structures, coupling to the quantized radiative electromagnetic field can no longer be neglected. In the next section, we discuss quantum-chemical applications in which treating the electromagnetic field as a dynamical quantum degree of freedom is essential for reproducing experimental observations. For this purpose it is necessary to consider the framework of nonrelativistic QED where matter and radiative dynamical EMF fields are mutually coupled.

## 5 Atoms and Molecules Coupled with Quantum Fields

Atoms and molecules are constantly subject to quantum vacuum fluctuations arising from all underlying fields, mainly the electromagnetic field, but also the Dirac electron-positron field. The fluctuating EMF interacts with matter and can lead to feedback mechanisms, whereby the field alters the properties of matter and matter responds by exciting the field. The strength of these interactions is determined by how the field–matter coupling compares with the relevant loss and decoherence rates in both subsystems, i.e., relaxation and dephasing of the material excitations and photon loss of electromagnetic modes. When the rate of coherent field–matter energy exchange is slower than these decay processes, the system is in the weak-coupling regime. Such interactions can be studied perturbatively by treating them as perturbations to the isolated field and matter systems. In contrast, when the energy exchange between field and matter becomes faster than the losses of the combined system, the matter-field system must be described by hybridized states,^90^ which can decouple after some time (e.g., in a cavity). Hence, the dynamics of each entity in the combined system become strongly interdependent, leading to hybrid states known as polaritons. The rapid, repetitive exchange of energy that occurs before energy leaks out of the system is called Rabi oscillation. Instead of the molecule’s original energy levels, the hybrid state splits into two distinct levels (Upper and Lower Polaritons), separated by a gap called the Rabi frequency.^51,52^ By combining quantum mechanical descriptions of the electromagnetic field, for example, as found in cavity quantum electrodynamics, with the complete molecular characterization based on potential energy surfaces used in chemistry, these hybrid states are studied. In such studies, the goal is to generalize well-developed concepts from quantum chemistry to field-matter hybrids.

The realization that fluctuating electromagnetic fields mediate molecular interactions has revealed new opportunities to tailor material behavior by controlling those fluctuations through spatial confinement or external fields. The electronic structure and properties of the molecules “dressed” by the fluctuating electromagnetic field in general deviate from those of the isolated molecules. To understand the physical concept of a dressed state, one can consider the paradigmatic shift in the energy levels of a hydrogen atom known as the Lamb shift. Although conceptually distinct, both the Lamb shift and polaritonic level restructuring originate from the coupling of matter to the quantized electromagnetic field and can be viewed as manifestations of electromagnetic dressing in different coupling regimes. Understanding such states requires a theoretical framework in which both the field and matter degrees of freedom are treated quantum-mechanically and evolve under the same fundamental principles. In this section, we discuss noncovalent and covalent interactions in QED and explain why a quantum electrodynamical extension of conventional quantum chemistry is essential for studying the mediation and modification of these molecular interactions via vacuum fluctuations.

### 5.1 Weak Matter-Field Coupling

Weak matter-field coupling is the fundamental mechanism behind numerous physical phenomena, including the Lamb shift and spontaneous emission. In molecular QED, intermolecular interactions are mediated through the vacuum electromagnetic field. Resonant energy transfer, vdW dispersion interactions, and scattering phenomena are examples of field-mediated interactions between atomic and molecular entities. In these cases, matter excitations are transferred to the electromagnetic field with probabilities that depend on the field’s density of states. Consequently, they are influenced by the environment through its impact on the density of states of the field. Therefore, an advantage of considering these interactions from the QED perspective is that any change in the environment in which the systems interact, or the application of external fields to atoms and molecules, can be accounted for by examining how these changes affect fluctuations of the vacuum electromagnetic field. For example, imposing macroscopic boundaries on a system induces reflections of the vacuum field from these boundaries, thus altering the distribution of the field’s modes and, consequently, modifying the molecular interactions. Such scenarios are the focus of macroscopic QED.^6^

On the other hand, variations in matter systems, particularly changes in the electric and magnetic properties of atoms or molecules, can also alter how these entities interact with the vacuum field, thereby influencing molecular interactions. For instance, since chiral molecules exhibit distinct interactions with electromagnetic fields compared to nonchiral ones, molecular interactions within chiral systems also differ. Another example is when interacting atoms are initially excited. In such cases, in addition to the vdW dispersion interaction, resonance contribution is also present due to the exchange of real photons between the molecules.^177,178^

To evaluate the interaction energy between atoms, one must solve the equation of motion for the entire matter-field system, which is challenging even in the simplest scenario of two interacting atoms. Consequently, approximate methods and simplified physical models are necessary to address this problem. Among the approximate approaches in the weak coupling regime, perturbation theory stands out as one of the most widely employed, within which atom-field and atom–atom couplings are considered perturbatively. However, this approach is only practical for a small number of atoms, thereby avoiding higher-order perturbations. In the case of two atoms, the states of the unperturbed system with *Ĥ*_0_ = *Ĥ*_atom_ + *Ĥ*_field_ (see Section 3.3 of the main text and Section S3 of the Supporting Information for the definition of *Ĥ*_atom_ and cv_field_ in the minimal-coupling formalism of QED) are expressed as the product states of the atoms and the field, i.e.

15 $$\left|\Psi^{\left(0\right)}\right\rangle =\left|\psi^{\left(0\right)}\left(r_{1}\right)\right\rangle \left|\psi^{\left(0\right)}\left(r_{2}\right)\right\rangle \left|n_{k_{1}\lambda_{1}},n_{k_{2}\lambda_{2},...}\right\rangle$$

where *n*_*k*_*i*_λ_*i*__ denote the occupation numbers of the field’s modes with frequency ω_*i*_ = *ck*_*i*_ and polarization λ_*i*_, and |ψ^(0)^(***r***_*i*_)⟩ are the eigenkets of isolated atoms. Using these states, perturbation calculations can be performed to determine the energy of the total system under the perturbation Hamiltonian (eq 8). As is well-known in quantum field theory and QED, the energy of the matter-field system is infinite due to the fact that the energy of the fluctuating field in its vacuum state is the sum of zero-point energy of an infinite number of modes, *E*_f_^(0)^ = ∑_*i*=1_^∞^ℏ*ck*_*i*_. Nevertheless, it is evident that the interaction energy between the two atoms, which is dependent on the interatomic distance *R*, is finite and can be obtained by renormalizing the energy of the interacting matter-field system with respect to the energy of the noninteracting system (*R* → ∞).

An elegant approach to perturbative evaluation of interaction energies in QED and quantum field theory is the use of Feynman diagrams. In the minimal-coupling formalism of molecular QED, typical Feynman diagrams representing the interaction of two atoms resemble those depicted in (Figure 1a).^179^ Vertices in these diagrams denote atom–atom or atom-field interactions accompanied by virtual transitions of the subsystems, thereby indicating the perturbation order for each scenario depicted in the diagram. Consequently, diagrams (i) and (ii) represent second-order perturbation, (iii) and (iv) represent third-order perturbation, and diagram (v) represents fourth-order perturbation. Diagram (i) illustrates an interaction between the atoms from second-order perturbation with the instantaneous dipolar Coulomb coupling *V*_dd_. This diagram corresponds to the same physical process responsible for London dispersion interaction in semiclassical theory. In contrast, in diagram (ii), the interaction is solely attributed to the term ***Â*** ^2^ and the exchange of two virtual photons that are emitted or absorbed simultaneously at the atomic centers. The third-order diagrams (iii) and (iv) depict interactions arising from mixed processes. In diagram (iii), an instantaneous dipolar Coulomb coupling (*V*_dd_) is followed by the exchange of a virtual photon between the atoms due to two consecutive atom-field couplings ***p̂*** · ***Â*** at the two atomic centers. The physical process underlying diagram (iv) involves the exchange of two virtual photons between the two atoms. In the first atom-field coupling (***p̂*** · ***Â***) at the atomic center *B*, a virtual photon is emitted. Subsequently, atom *A* interacts with the field via ***Â*** ^2^, which involves simultaneous emission and absorption of two virtual photons [Note that ***Â*** ^2^ includes combinations of creation and annihilation operators of photons (*â*_*n*_^†^ and *â*_*n*_, respectively) such as *â*_*n*_^†^ *â*_*n*_ and *â*_*n*_ *â*_*n*_^†^, enabling two-photon processes through ***Â*** ^2^ atom-field couplings. For more details about these bosonic operators and their commutation relations, refer to Section S1 of the Supporting Information]. Finally, the remaining photon is absorbed in another ***p̂*** · ***Â*** interaction at atom *B*. The fourth-order diagram (v) is the result of four consecutive ***p̂*** · ***Â*** interactions (two at each atom), which lead to the exchange of two virtual photons between the atoms.

Since the dipole polarizability of an atom is proportional to *e* ^2^, or equivalently to the dimensionless fine-structure constant α, the power of *e* ^2^ or α can be regarded as a measure of the magnitude of atom–atom and atom-field interactions. Considering the order of perturbations and the physical processes associated with each diagram in (Figure 1a), it is evident that all five diagrams exhibit the same power of *e* ^4^ (or α^2^), indicating that they possess comparable physical significance, despite originating from different perturbation orders. Naturally, there are additional diagrams, but all of them can be classified into one of these five categories. By summing over all possible diagrams, the total interaction energy between the two atoms can be determined. Including all possible diagrams is essential to ensure that the interaction energy is computed from a physical picture consistent with QED principles. In particular, in diagrams (i) and (iii), the two atoms interact via an instantaneous dipolar Coulomb coupling, which, at first, might seem unphysical within the QED framework. However, considering every relevant contribution of the same order with respect to the fine structure constant (α), all instantaneous contributions to the interaction energy cancel out,^33^ and the remaining expression is fully retarded, thereby complying with the relativistic nature of the fluctuating electromagnetic field. This cancellation of instantaneous interactions indicates that, in molecular QED, all interactions are effectively mediated through the field and the exchange of transverse virtual photons.

The mediation of intermolecular interactions by the fluctuating electromagnetic field explains the retardation effects on these interactions. For two atoms to interact, they must interact with the field and borrow polarization and energy from it in the form of virtual transitions. Then, the borrowed polarization and energies enable the atoms to interact via the exchange of virtual photons. However, the borrowing process is subject to the uncertainty principle. The borrowed energy must be returned within a time frame that does not violate the energy-time uncertainty. This means that, at large interatomic distances, where virtual photons have to travel for a long time, those virtual photons belonging to the electromagnetic field’s modes with higher frequencies contribute less compared to those with lower frequencies. Conversely, high-frequency modes contribute more significantly to the interaction when the interatomic distance is small.^4^ In the nonretarded limit, where the interatomic distance *R* is significantly smaller than the characteristic atomic wavelengths, i.e., *R* ≪ λ, the general expression for the interaction energy can be approximated to reproduce the London dispersion interaction (*R* ^–6^). In the opposite limit, where *R* ≫ λ, the interaction energy is given by the Casimir-Polder expression (*R* ^–7^).^33^

An alternative formalism of molecular QED can be obtained by applying a quantum canonical transformation (the PZW transformation), to the minimal-coupling Hamiltonian (eq 8). As shown in Section S3 of the Supporting Information, in the resulting Hamiltonian, matter-field interactions arise from the coupling of matter’s electric and magnetic multipole moments to the electric and magnetic fields; hence, the formalism is referred to as multipolar coupling. In dipole approximation, the interaction Hamiltonian is given by *Ĥ*_int_^mult^ = −∑_ξ_x3b5_0_^–1^ **μ̂**_ξ_· ***D* ^**^⊥^(***R***_ξ_), where **μ̂**_ξ_ is the electric dipole moment of atom ξ and ***D* ^**^⊥^ is the transverse component of the microscopic displacement field (there is also another term in the multipolar Hamiltonian that must be taken into account when computing self-energies, e.g., Lamb shift, but does not play a role in the interaction between atoms or molecules^4,5^ ). In this formalism, the interaction Hamiltonian consists solely of couplings between matter and the field, with no direct couplings among the matter components; therefore, the retarded nature of the interactions is evident from the Hamiltonian. Additionally, the multipolar form of matter-field interactions facilitates the interpretation of the physical behavior of the resulting interaction energies, as they have counterparts in classical electromagnetism. It is seen that in multipolar formalism, the dispersion interaction between two neutral nonpolar atoms is obtained from fourth-order perturbation theory with the interaction Hamiltonian *Ĥ*_int_^mult^.^4,5^

As noted earlier, one advantage of the QED perspective on intermolecular interactions is that it enables us to understand how physical conditions influence these interactions by considering their effects on the quantum-mechanical fluctuations of the electromagnetic field. Introducing media or boundaries, such as macroscopic bodies, disrupts and alters these fluctuations. Scattering of the electromagnetic field at these boundaries alters the distribution of modes and their fluctuations, leading to new interactions between atoms. In macroscopic QED, these medium-induced effects are analyzed using classical Green’s functions that incorporate all the geometric and electromagnetic properties of the environment in which the atoms interact.^6^

An alternative approach to modifying the fluctuating electromagnetic field is to apply an external dynamic field, such as a monochromatic laser beam. This external field can be regarded as an excitation of the vacuum field, thereby producing real photons in addition to the virtual photons that atoms use for interaction. In an intense field, dispersion interactions undergo significant changes and scale with the interatomic distance *R*, unlike the well-known Casimir-Polder and London dispersion energies. These changes depend on the polarization of the applied field and the orientation of the interacting pair relative to the direction of the field’s propagation. When averaged over all directions of ***R***, an attractive dispersion interaction ∝*R* ^–1^ is observed in the near zone. Conversely, in the far zone, the dispersion interaction is proportional to *R* ^–2^ *k*_f_ sin­(2*k*_f_ *R*), where *ck*_f_ represents the frequency of the applied field. Consequently, the interaction in the far zone can be either attractive or repulsive depending on the ratio *k*_f_/*R*.^26^

Instead, if the external field is static, the dynamics of the electromagnetic field remain unchanged, but the atoms acquire additional static dipole moments that allow new channels of interaction with the vacuum field and with one another. In the multipolar-coupling scheme, this corresponds to extending the interaction Hamiltonian to *Ĥ*_int_^mult^ = −∑_ξ_x3b5_0_^–1^[**μ̂**_ξ_^(*f*)^ + **μ̂**_ξ_^(*s*)^]· ***D* ^**^⊥^(***R***_ξ_), where **μ̂**_ξ_^(*s*)^ is the external-field–induced dipole and **μ̂**_ξ_^(*f*)^ the intrinsic fluctuating dipole. Perturbation theory then yields interaction-energy contributions Δ*E* = Δ*E*_*ss*_^(2)^ + Δ*E*_*fs*_^(4)^ + Δ*E*_*ff*_^(4)^ + ..., where each term corresponds to a distinct atom–field coupling channel. The *ss* channel describes the interaction of two induced dipoles mediated by the vacuum field, giving a field-induced electrostatic term ∝*R* ^–3^ that may be attractive or repulsive. The mixed *fs* channel corresponds to the coupling of a fluctuating dipole on one atom with an induced dipole on the other, producing an always-attractive polarization interaction ∝*R* ^–6^. Finally, the *ff* channel represents the coupling of two fluctuating dipoles and produces the familiar dispersion interaction ∝*R* ^–6^ (nonretarded) or ∝*R* ^–7^ (retarded). The many-body character of these forces and the interplay between them imply that external static fields can be used to tailor noncovalent interactions (for example, in a benzene dimer as shown in (Figure 5a)),^180–184^ enabling applications such as tunning materials’ properties using external fields^185–187^ and field-assisted exfoliation of layered nanostructures.^188,189^

**Figure 5:**
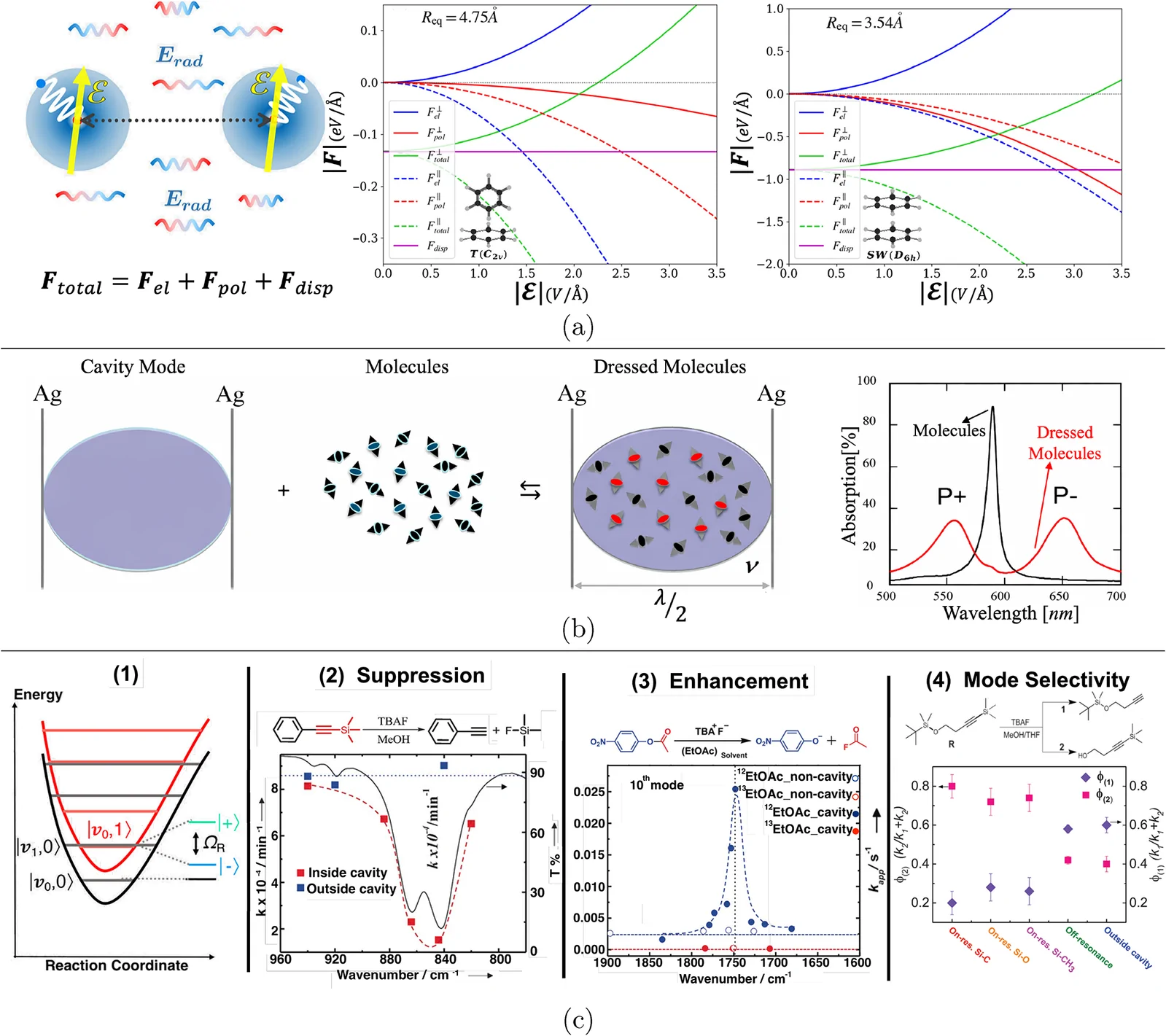
Atoms and Molecules Coupled with Quantum Electromagnetic Field (EMF): (a) interplay between molecular forces induced by an external static field and dispersion force due to the vacuum EMF in two configurations of a benzene dimer demonstrates the possibility of tailoring molecular forces through external fields. Many-body characteristics of dispersion and field-induced polarization forces (taken into account using the MBD method^85^) result in an intricate interplay between these forces and the field-induced electrostatic force. Reproduced from ref 181. Available under a CC-BY 4.0 license. Copyright 2022 M. R. Karimpour, D. V. Fedorov, and A. Tkatchenko. Published by American Chemical Society. (b) Electronic strong-coupling of molecules with the cavity EMF yields the matter-field hybridization and formation of upper and lower polaritons that are spectroscopically observable. Reproduced from ref 51. Copyright 2016 American Chemical Society. (c) Vibrational strong-coupling of molecules with the cavity EMF: (1) formation of vibrational polariton states | ± ⟩from hybridization of molecular and cavity excitations. (2) Suppression of reaction rates as a function of cavity photon frequency inside and outside the cavity, with the IR spectrum of the uncoupled molecule. (3) Enhancement of reaction rates under vibrational strong coupling. (4) Cavity-induced mode selectivity in a reaction with two competing products. Reproduced from ref 52. Available under a CC-BY 4.0 license. Copyright 2023 A. Mandal, M. A.D. Taylor, B. M. Weight, E. R. Koessler, X. Li, and P. Huo. Published by American Chemical Society. [Panel (c.2) is reproduced with permission from ref 63. Copyright 2016 John Wiley and Sons. Panel (c.3) is reproduced with permission from ref 65. Copyright 2019 John Wiley and Sons. Panel (c.4) is reproduced with permission from ref 64. Copyright 2019 The American Association for the Advancement of Science.].

In addition to these external influences, the structure of the matter system itself plays a crucial role in the resulting QED effects, since molecular vdW interactions inherently reflect the collective response of many atoms to vacuum fluctuations. These interactions cannot be obtained by summing pairwise contributions^85^ because each atom couples not only to the vacuum field but also to the fluctuating dipoles of its neighbors. As more atoms or molecules are added, the pattern of electromagnetic fluctuations changes, which alters both the interactions among particles and the overall coupling of the system to the quantum vacuum field. Many-body effects are known to delocalize the charge density along symmetry axes in extended systems such as macromolecules and nanostructures,^84^ leading to substantial enhancements in atomic polarizabilities and, consequently, atom-field couplings. The resulting increase in matter-field coupling amplifies QED effects and makes explicit many-body dispersion essential in any realistic QED description of large molecular assemblies. Since solving the fully coupled atom–field dynamics is generally implausible, practical treatments rely on controlled approximations, numerical many-body methods, and simplified models, such as a QED extension of MBD.^43,190^

Although research on intermolecular interactions in the weak-coupling regime has been ongoing for decades, many fundamental aspects remain unknown and debated. For instance, how external static fields can (or cannot) influence intermolecular interactions mediated through the fluctuating vacuum EMF^180,182,183,191–194^ by altering the response properties of atoms and whether these changes have classical or quantum mechanical characteristics? Another crucial question is how retardation affects these interactions at intermediate intermolecular distances, comparable to the atomic time scale, which cannot be simply categorized as near- or far-zone. This question was recently considered by Brambilla et al. using an effective field theories approach for two hydrogen atoms,^195^ where they also demonstrated that there is a correction to the London dispersion at short ranges that scales with the interatomic distance as *R* ^–3^. Although this correction resembles an electrostatic term, it is a quantum mechanical correction arising from loop diagrams. Such corrections suggest that the usual truncation of interactions at the leading dipole–dipole order may not accurately represent the physical picture of intermolecular interactions, even for a simple dimer, and particularly when the interactions are mediated through the fluctuating EMF.^43^ These examples, along with many others, indicate that we have only scratched the surface of our understanding of intermolecular interactions from the first-principles of QED.

### 5.2 Strong Matter-Field Coupling

In contrast to the weak-coupling regime, where matter-field interactions can be treated perturbatively, strong matter-field couplings render QED effects highly influential and beyond the validity of perturbation theory. In the strong-coupling regime, matter and the quantized field must be treated as a hybrid system, as explored in polaritonic chemistry. This approach aims to understand how strong interactions between matter and the quantized field within optical or plasmonic cavities or circuit QED devices modify molecular structure, dynamics, and reactivity,^55,56,62,63,65,93,95^ as illustrated, for example, in (Figure 5b,c). However, nonperturbative treatments of such hybrid systems are challenging and often intractable, even for small molecules. Therefore, the introduction of simpler, more intuitive model Hamiltonians, along with various approximations, is necessary to simplify these complex matter-field systems. Yet, given the extensive literature on strong matter-field coupling, no single model is universally adopted. Instead, a variety of Hamiltonians, each differing in technical aspects, is employed.^52^

The introduction of various Hamiltonians is primarily driven by the need to balance intuitive understanding with rigorous accuracy across different regimes of light-matter coupling.^29,52,196^ Simple models like the Jaynes-Cummings (JC) and Tavis-Cummings (TC) Hamiltonians are frequently employed because they offer analytic solutions and provide clear physical insights, such as the scaling of Rabi splitting with the number of molecules (√*N*).^29,56^ However, these models rely on approximations, specifically the rotating wave approximation and the neglect of dipole self-energy, which become inadequate in the ultrastrong coupling regime or when describing realistic molecular potential energy surfaces.^29,197–200^ Consequently, more rigorous models, such as the Pauli-Fierz (PF) Hamiltonian, are essential to ensure ground-state stability and accurately capture the physics when simpler models fall short.^52,53^ In addition, distinct Hamiltonian forms are required to bridge the gap between detailed ab initio simulations of individual molecules and the collective mechanics of macroscopic ensembles observed in experimental cavities.^56,196,201–203^

Furthermore, the variety of Hamiltonians arises from the mathematical freedom to choose a gauge in QED and the practical constraints of computational simulations. While the Coulomb gauge (***p* ^**· ***A* ^**) and Dipole gauge (**μ̂**· ***E* ^**) are theoretically equivalent in a complete basis, they yield conflicting numerical results when the electronic basis is truncated, a problem known as “gauge ambiguity”.^52^ This necessitates the development of properly truncated Hamiltonians to ensure physical consistency. Additionally, the choice of Hamiltonian often depends on the specific computational strategy employed. For instance, parametrized QED (pQED) employs precomputed molecular states/potentials as fixed input parameters to enhance computational efficiency, while self-consistent QED (scQED) integrates the photon field into the electronic-structure calculation, necessitating a Hamiltonian that permits variational relaxation of molecular orbitals.^24,31,52,196,199^

As in the case of weak coupling, the starting point for deriving QED from first-principles is the minimal-coupling Hamiltonian in Coulomb gauge. The interactions of charged particles in the matter system with the quantized electromagnetic field, as described by the principle of minimal coupling of electromagnetism, are incorporated into the system’s kinetic energy. Since the particle’s momentum operator operates nonlocally in the energy space and couples states that are far apart in energy, solutions obtained from this model are often more difficult to converge numerically. In contrast, the multipolar-coupling scheme of molecular QED, derived from the minimal-coupling Hamiltonian through the PZW unitary transformation, expresses matter-field interactions in terms of the couplings of matter dipole (or generally multipole) moments to the transverse electric field.^29,52,53,198^ Since the dipole moment is local in energy space, this gauge is numerically more stable and accurate for truncated basis sets compared to the Coulomb gauge.

Strong coupling between molecular electronic transitions and the quantized electromagnetic field gives rise to hybrid states known as upper and lower polaritons, separated by the Rabi splitting energy, as shown in (Figure 5b). This hybridization creates a distinct spectroscopic splitting in the absorption peak, observable even under vacuum-field conditions without external laser excitation,^52,62^ and is particularly pronounced in organic molecules with large transition dipoles.^55,56^ Fundamentally, this interaction reshapes the system’s energy landscape, replacing the Potential Energy Surfaces (PES) of the isolated molecule with distinct Polaritonic Potential Energy Surfaces (PoPES).^62^ This reshaping can induce new dynamical features, such as cavity-induced conical intersections, which allow molecules to transition rapidly between energy states. These intersections can guide molecular geometry toward specific products or, through the accumulation of Berry phase shifts, generate destructive interference that effectively blocks specific reaction paths.^62^ Alternatively, because the lower polariton lies energetically below the uncoupled excited state, it can act as a trap that favors relaxation back to the ground state rather than the forward reaction. This energetic stabilization can suppress the reaction rate, thereby increasing the stability (yield) of the starting reactant.^52,56,62,93^ Therefore, electronic strong coupling can fundamentally alter reaction kinetics, pathways, and yields. Hence, in theory, by tuning the cavity frequency, the potential energy surfaces can be engineered to favor one product over another, leading to highly selective isomerization.^52^

The creation of polaritons in a system comprising numerous molecules can occur through collective strong coupling between matter and the quantized field of a cavity. In such scenarios, the hybrid states become delocalized across many molecules, thereby enhancing the material’s transport properties and altering its work function. Consequently, this leads to an increase in the material’s conductivity and has implications for device performance in applications such as organic light-emitting diodes, photovoltaics, and molecular electronics.^24,51,52,56,62^

In addition to the electronic degrees of freedom of molecules, coupling the cavity mode to molecular vibrational transitions can give rise to new hybrid field-matter states known as vibro-polaritonic states.^52,63,65,204^ Vibrational Strong Coupling (VSC) targets specifically the molecular vibrations (bond stretching and bending) that occur naturally in the electronic ground state. Unlike electronic strong coupling, which requires high-energy photons to excite electrons into excited states, VSC operates in the infrared regime, where vibrational energy levels are accessible via thermal fluctuations. By coupling to these vibrations, the cavity modifies the potential energy landscape that the molecule navigates while in its ground state. The mechanism behind this modification is often described as dynamical caging.^52,95^ The cavity photon mode behaves like an additional solvent environment attached to the reacting bond, thereby increasing friction on the reaction coordinate. This can trap the molecule near the transition-state barrier, suppressing the reaction rate, or modify the flow of vibrational energy between different parts of the molecule, also affecting the reaction rate (see Figure 5c). Since these effects directly influence the vibrational motion, they alter the reaction rate without requiring the molecule to leave its ground electronic state.^51,52,63,95^

### 5.3 Quantum Electrodynamical DFT

QEDFT is an exact reformulation of the underlying theory of nonrelativistic QED derived from the Pauli-Fierz Hamiltonian in the Coulomb gauge, specifically designed to be computationally feasible for complex systems.^34^ The theoretical foundation of QEDFT lies in a powerful mapping theorem, analogous to the Hohenberg–Kohn and Runge-Gross theorems of standard DFT and time-dependent DFT (TDDFT).^205^ This theorem states that the complete state of a coupled electron-photon system, represented by the full matter-field wave function Ψ­(***r***_*j*_, *q*_α_, *t*), is uniquely determined by a much simpler set of basic variables. These variables include the time-dependent electronic density *n*(***r***, *t*) and the expectation values of the photonic oscillators’ coordinates *q*_α_(*t*), which correspond to the displacement field of each cavity photon mode α.^29,34,35,205^ This profound simplification implies that, in principle, the computationally intensive task of computing the complex many-body wave functions can be avoided. Instead, all the properties of the system can be derived from these two, significantly lower-dimensional quantities.

To make this mapping practical, QEDFT uses the Kohn–Sham (KS) construction. It replaces the real, interacting system of electrons and photons with a fictitious, noninteracting system that reproduces the same basic variables *n*(***r***, *t*) and *q*_α_(*t*). This replacement leads to self-consistent Maxwell-Kohn–Sham equations, including a set of single-particle Schrödinger-like equations for the KS electronic orbitals ϕ_*i*_(***r***, *t*) with an effective potential *v*_KS_(***r***, *t*) = *v*_ext_(***r***, *t*) + *v*_H_(***r***, *t*) + *v*_pxc_(***r***, *t*) where the new term *v*_pxc_(***r***, *t*) collects all photon-mediated mean-field plus exchange–correlation effects on electrons, and a set of coupled harmonic oscillator equations for the photon coordinates *q*_α_(*t*) driven by the electronic dipoles of the matter.^34,35^ Crucially, these equations are coupled, as the electronic density acts as a source for the photon field, and the photon field acts back on the electrons, creating a self-consistent feedback loop that is absent in standard TDDFT, where the light field is a fixed external input.^196^

The connection between the real and KS systems lies in the effective KS potential *v*_KS_(***r***, *t*), which incorporates the conventional terms from electronic DFT (external, Hartree, and electron–electron exchange-correlation) along with the novel photon exchange-correlation (pxc) potential *v*_pxc_. This term encompasses all nonclassical many-body effects arising from electron-photon interactions, including electron self-interaction mediated by emitted photons and other correlations. Consequently, in addition to the conventional density–density response function χ_*nn*_, the formalism yields cross-response functions χ_*nq*_ and χ_*qn*_, as well as the photon–photon response χ_*qq*_. This extended response matrix has two significant practical implications. First, electronically excited states manifest as perturbations of the quantized photon field, redefining spectroscopy both conceptually and computationally, from which polaritonic excitations, intrinsic radiative lifetimes, and spectral line widths emerge naturally without the need for phenomenological reservoirs or damping terms. Second, a direct generalization of the random-phase approximation (RPA), termed polaritonic RPA (pRPA), efficiently captures key cavity-mediated phenomena. Moreover, the inclusion of nonclassical electron–photon exchange–correlation kernels allows the theory to describe multiphoton processes and higher-order light–matter correlations that become important in the strong-coupling regime.^28,198^

Because the exact form of *v*_pxc_ is unknown, the primary challenge in applying QEDFT is finding suitable approximations for the photon exchange-correlation functional. A practical approximation scheme developed for QEDFT is based on the Optimized Effective Potential (OEP) method.^31,206^ The core idea is to derive the potential from an orbital-dependent energy functional. In the lowest order of perturbation theory, the electron-photon exchange energy corresponds to the Lamb shift of the ground state energy. Reformulation of the OEP equations using the Sternheimer equation avoids the need to calculate unoccupied electronic states,^31,207^ which is computationally prohibitive, and instead requires only the occupied orbitals. This development enabled the first ab initio QEDFT calculations on real three-dimensional molecules.^31^ The accuracy of this exchange-only OEP has been benchmarked against exactly solvable models, demonstrating that it performs well for both weak and strong couplings, except in specific cases of near-degeneracy where multiphoton processes become dominant.^31,206^

Although the OEP provides a prominent approximation to the pxc functional, it is not universally applicable and can fail in certain physical situations.^208^ The OEP functional, by construction in its lowest order, primarily accounts for single-photon processes.^31,206^ Therefore, when multiphoton processes dominate in the strong-coupling regime, and the ground state becomes nearly degenerate with the first excited state, the OEP approximation breaks down because it does not capture higher-order multiphoton processes. Besides that, even when the QEDFT calculation for the basic variables (*n*(***r***, *t*) and *q*_α_(*t*)) is accurate, calculating other physical observables still requires approximations for those observables’ functionals, which is challenging on its own.^24,198,200^

Despite its formal elegance, ab initio QEDFT is computationally demanding, much like conventional DFT, and is therefore limited to small systems. This contrasts with most polaritonic chemistry experiments, in which strong coupling is achieved by having many molecules interact simultaneously with a single cavity mode.^51,52^ As a result, QEDFT studies typically simulate only a single molecule and often artificially increase the light–matter coupling to reproduce observable cavity effects.^95^ These simplifications reduce statistical reliability and leave unresolved the connection between single-molecule simulations and many-molecule experiments. A further limitation is that most QEDFT implementations treat cavity modes as lossless, whereas real cavities exhibit leakage and absorption.^27,35,95,196,198,202^ A practical approach is to combine QEDFT for the electronic subsystem with macroscopic QED for the photonic environment,^6,202^ which incorporates dissipation through Green’s tensors and fluctuation–dissipation relations. Developing efficient hybrid schemes of this kind remains an active area of research.

In conclusion, the mapping theorem at the core of QEDFT generalizes the highly successful principles of DFT to systems where electrons and photons are treated on equal quantum footing. This formally exact framework provides a rigorous foundation for building systematic approximations, distinguishing it from model-based cavity QED approaches. Recent developments, including relativistic and linear-response formulations of QEDFT,^38,78^ have extended its reach to realistic molecules and materials, while polaritonic response theory offers new tools for computing optical observables.^53^ Moreover, hybrid schemes that combine density-functional and macroscopic QED formalisms^202^ demonstrate how QEDFT can be embedded in lossy or structured photonic environments, thereby bridging the microscopic and continuum scales.

Nevertheless, the practical accuracy of QEDFT still depends critically on the development of reliable electron–photon exchange–correlation functionals and on the validation of predictions under realistic experimental conditions.^52,201,208^ Cavity-induced ground-state modifications may be overstated if losses, multimode fields, and disorder are not properly accounted for, and collective or thermal effects can often mimic purely quantum-vacuum signatures.^196,201^ These considerations underscore the necessity for cross-validation with complementary ab initio frameworks, such as QED-coupled-cluster or hybrid DFT+macroscopic-QED approaches, to ensure that QEDFT achieves both conceptual rigor and quantitative reliability in describing cavity-modified molecular and material properties.

## 6 Precision Spectroscopy

Precision physics and spectroscopy enable the measurement of atomic and molecular properties with exceptional accuracy, often reaching 10 to 15 decimal places. The primary objective is to use atoms and molecules as microscopic laboratories to test fundamental physical laws.^79^ For instance, precision measurements of the hydrogen 1*S*–2*S* transition have tested QED to 15 decimal places.^209^ Such experiments advance scientific understanding by verifying the Standard Model and QED, refining fundamental constants, and providing opportunities to identify new physics when discrepancies arise between theoretical predictions and experimental results.^79,82,91,92,210^ To achieve theoretical results that match the experimental precision, one must employ a framework that incrementally constructs the system’s energy. Such a framework can be based on nonrelativistic QED (nrQED) or relativistic QED (rQED).^211^ The construction of both nonrelativistic and relativistic QFTs is briefly discussed in Sections S1 and S2 of the Supporting Information. Interested readers are referred to consult the references provided in these sections.

The starting point is to calculate a base energy for the atom or molecule. In the standard Born–Oppenheimer approach, nuclei are assumed to be stationary, and the Schrödinger equation is solved for the electrons. In contrast, the pre-Born–Oppenheimer approach^81,82^ treats all particles as quantum entities and solves the equation without fixing the nuclear positions. Although this method is computationally intensive, it eliminates errors arising from the assumption of stationary nuclei, yielding more accurate base energies and equations. Then, to obtain the true energy, theoretical models incorporate small corrections to the base equations. These corrections are typically expressed as a series expansion in terms of the fine-structure constant, which quantifies the strength of the electromagnetic interaction.

The framework incorporates several effects, including nonadiabatic, relativistic, and QED corrections.^91^ Nonadiabatic corrections account for nuclear motion and its interaction with electrons, thereby relaxing the assumption of stationary nuclei in the Born–Oppenheimer approach. Relativistic corrections account for mass-velocity effects and electron spin, which become significant as electrons approach the speed of light.^79,91^ QED corrections, which are the most complex to compute, address quantum field theory phenomena such as self-energy, vacuum polarization, and retardation.^79,81,211^ When starting from a nonrelativistic equation and adding large corrections, these adjustments can be inefficient and susceptible to mathematical divergence. Consequently, studies recommend beginning with a relativistic wave equation, such as the Bethe-Salpeter or Salpeter-Sucher equation, and employing the Dirac equation generalized for systems with two or more particles.^79,82,211^ This strategy incorporates relativity as an intrinsic aspect of the system, thereby reducing computational demands and potentially improving accuracy for heavy atoms where relativistic effects are pronounced.

The Bethe–Salpeter (BS) equation serves as the fundamental field-theoretic starting point for describing a bound state as a pair of particles undergoing an infinite number of scattering events over an infinite lifetime. Because the full BS equation is challenging to solve for atoms and molecules, it is transformed into an exact equal-time variant by integrating over the relative energy. This process yields the Salpeter–Sucher exact equal-time equation, which is formally exact and depends only on the particles’ spatial coordinates, making it compatible with traditional computational techniques. However, this equation remains nonlinear in energy due to terms that account for retardation and radiative effects. To obtain a practical wave equation, the nonlinear Salpeter–Sucher equation is partitioned. By neglecting the nonlinear QED terms, the no-pair Dirac–Coulomb–Breit (DCB) equation emerges as the dominant, linear, and Hermitian part. The framework first solves the no-pair DCB equation, accounting for special relativity and the dominant instantaneous interactions (Coulomb and Breit forces) to all orders in the fine-structure constant. Effects such as retardation, corrections due to the creation of virtual electron-positron pairs, and radiative corrections (self-energy) are subsequently added as perturbations to this relativistic reference.

A primary contribution of precision spectroscopy is the ongoing validation of QED as the dominant theory for matter–electromagnetic field interactions. Precision spectroscopy of light systems such as hydrogen (H) and helium (He) has provided stringent tests of QED.^79^ Non-Relativistic QED has been the ”golden standard” for light elements, yielding high-precision theoretical values that closely match experimental transitions. In systems such as the HD molecule, precision measurements have achieved sub-MHz uncertainty, revealing discrepancies of 1.4 to 1.9 σ (*std*) between calculation and experiment.^92^ These discrepancies indicate that current nonadiabatic and higher-order QED effects are not yet fully understood.

Precision physics has also revealed a significant discrepancy in measuring the proton’s size, known as the *proton-size puzzle*.^212–214^ By comparing transition frequencies in electronic hydrogen and muonic hydrogen, it was found that the derived proton charge radius differs significantly depending on whether an electron or a muon orbits the nucleus. These experiments are critical for refining the Rydberg constant and determining accurate nuclear charge radii.

Spectroscopy is increasingly employed to define physical units and constants, rather than just to observe them. Precision measurements of the ^4^He_2_^+^ molecular ion are used to determine the polarizability of the helium atom, which is essential for a proposed new pressure standard based on the number density of helium gas atoms.^91,215^ Additionally, experiments with muonium (Mu)^216^ have provided insights into the muon’s anomalous magnetic moment, a key area where potential new physics beyond the Standard Model may exist.

Precision measurements of positronium (Ps),^216^ an atom composed of an electron and its antiparticle, are used to test QED without the complexities introduced by nuclear structure. Ps is also a candidate for testing the equivalence principle of gravity on antimatter. For elements with atomic numbers greater than 50, precision mass spectrometry has enabled the investigation of electron binding energies through energy-mass equivalence, thereby testing relativistic QED in high-field regimes.^79^

Molecular rovibronic mapping, which characterizes the interplay of rotational, vibrational, and electronic energy levels, is fundamental to precision physics because it enables stringent tests of the Standard Model. Measuring these transitions with extreme accuracy allows for direct comparison between experimental data and QED-based calculations. The complex energy landscapes of small molecules have been mapped to unprecedented precision. For instance, in the hydrogen molecule, rovibronic states have been measured and calculated with parts-per-billion accuracy.^81^ For helium ion rotations, the lowest-energy rotational interval of ^4^He_2_^+^ was precisely determined to be 70.937589 cm^–1^, establishing a new benchmark for three-electron molecular systems.^91^

## 7 Concluding Remarks

When transitioning from standard Quantum Matter Theory (QMT) to frameworks that explicitly incorporate Quantum Field Theory (QFT) or Quantum Electrodynamics (QED), the computational cost depends strongly on the chosen approximations and the specific physical regime targeted. It is a common misconception that adopting a QFT framework inherently increases computational demands; in reality, field-theoretical approaches can sometimes drastically reduce the computational bottleneck of standard atomistic QMT.

For explicit wave function methods, quantizing the electromagnetic field does indeed severely increase computational costs. In polaritonic chemistry, ab initio wave function methods (such as QED-Full CI or QED-Coupled Cluster) require tensoring the standard electronic Hilbert space with the bosonic Fock space of the cavity photons. Even if the photonic space is truncated, this multiplicative expansion of the dynamical degrees of freedom exacerbates the inherent exponential scaling of standard QMT. However, treating the field does not strictly mandate this Fock-space explosion. Quantum Electrodynamical Density Functional Theory (QEDFT) elegantly bypasses this by mapping the interacting matter-photon system onto an effective Kohn–Sham system. Consequently, QEDFT retains a formal scaling comparable to standard electronic TDDFT (typically *O*(*N* ^3^) to *O*(*N* ^4^)), allowing strong-coupling phenomena to be computed for relatively large molecules.

Crucially, in the macroscopic regime, field-theoretic approaches provide a profound computational advantage by enabling the exact reformulation of the system in terms of collective or macroscopic properties. When calculating long-range dispersion forces over large supramolecular distances, correlated-atomistic QMT methods such as CCSD­(T) scale as *O*(*N* ^7^) and quickly become intractable. In contrast, Macroscopic QED provides a highly efficient alternative by coarse-graining the system. Within this formalism, massive many-particle systems can be treated as macroscopic objects characterized entirely by well-defined electromagnetic response functions, such as bulk dielectric permittivities. The atomistic details are rigorously integrated out, and the physical interactions (such as Casimir-Polder forces) are computed by evaluating how these macroscopic boundaries scatter the quantized fluctuating electromagnetic field.

Even when discrete molecular representations are retained, QFT-inspired frameworks such as the Many-Body Dispersion (MBD) model map the continuous matter polarization field into a system of coupled quantum harmonic oscillators, capturing collective correlations with highly favorable *O*(*N* ^3^) scaling. Thus, while explicit photon counting at the microscopic level is computationally daunting, the mathematical flexibility of a field-theoretic standpoint is often precisely the tool needed to drastically reduce degrees of freedom and improve scalability for large systems.

Ultimately, the application of quantum field theory techniques to chemical systems is an emerging and highly promising field of research. Hence, one expects to have only scratched the surface of the plethora of effects and approaches that can aid our understanding of the complex behavior of many-body matter-field chemical systems. QFT methods are poised to substantially enhance the arsenal of quantum-chemical techniques while also highlighting the vacuum fields as dynamical quantum entities. QFT may also help to interpret and visualize complex many-body states as fields in real space, and yield novel scaling laws for the quantum properties of atoms, molecules, and materials. Connections to the broad and rapidly developing research fields of quantum information theory and quantum computing are also enabled by QFT-inspired tools and language. We thus encourage developers and practitioners of quantum chemistry to dive deeply into QFT-inspired methodologies and embrace the QFT-driven path for the further development of chemical theory.

## Supplementary Material



## Notes

Short QFT primer covering general aspects of QFT, representations of the canonical (Anti)­commutation relations, construction of non-relativistic QFTs, construction of relativistic QFTs, interacting fields and physical quasi-particle fields; brief introduction to the dirac free equation; molecular quantum electrodynamics in a nutshell (PDF)
