# Supplementary material for: Quantum Field Approaches to Chemical Systems
Source: J Am Chem Soc. 2026 Sep 7;148(37):39379–404. doi: 10.1021/jacs.6c02077 (PMC13614208; doi:10.1021/jacs.6c02077)
Supplement: Supplementary file 1 [file ja6c02077_si_001.pdf]

Supporting Information for

Quantum Field Approaches to Chemical Systems

Reza Karimpour, Matteo Gori, and Alexandre Tkatchenko*

Department of Physics and Materials Science, University of Luxembourg, L-1511 Luxembourg City, Luxembourg

E-mail: alexandre.tkatchenko@uni.lu

S1 A Short QFT Primer

S1.1 General Aspects of QFT

At its core, QFT treats each field as a quantized entity whose mode amplitudes—when expanded over a complete set of square-integrable, normalized basis functions (the single-particle modes)—become operators acting on a Hilbert space. The spectrum of the corresponding number operators is discrete and labeled by natural numbers, which define the occupation number of a given mode, i.e., they "count" the number of particles in that state. This procedure, commonly known as *second quantization*, provides a bridge between classical field amplitudes and the discrete quantum description of matter and radiation. For completeness, we briefly summarize the essential formalism here, referring the reader to these references 1–3.

As in any quantum theory, quantum field theory is based on the specification of a triad:

- An algebra of operators $\mathfrak{A}$, within which a subset of Hermitian operators is identified as the *observables*;

- A Hilbert space $\mathcal{H}$ on which the operators in $\mathfrak{A}$ act;
- A Hermitian Hamiltonian operator $\hat{H} \in \mathfrak{A}$ that generates the time evolution of observables, i.e.,

$$i\hbar \frac{\mathrm{d}\widehat{A}}{\mathrm{d}t} = [\widehat{A}, \widehat{H}] \,. \tag{S1}$$

The fields subjected to the second-quantization procedure include the wavefunctions associated with the fundamental matter degrees of freedom and the vector potential of the electromagnetic field (EMF).

The core of field quantization consists in defining an algebra of creation and annihilation operators $\widehat{a}(\boldsymbol{s})$ and $\widehat{a}^{\dagger}(\boldsymbol{s})$ for a set of modes, typically identified with a complete infinite orthonormal set of single-particle wavefunctions labeled by $\boldsymbol{s}$. The bosonic (fermionic) nature of these operators is encoded in the canonical (anti)commutation relations

$$[\widehat{a}_{\boldsymbol{s}}, \widehat{a}^{\dagger}_{\boldsymbol{s}'}]_{\pm} = \delta_{\boldsymbol{s}\boldsymbol{s}'} \,, \qquad [\widehat{a}_{\boldsymbol{s}}, \widehat{a}_{\boldsymbol{s}'}]_{\pm} = [\widehat{a}^{\dagger}_{\boldsymbol{s}}, \widehat{a}^{\dagger}_{\boldsymbol{s}'}]_{\pm} = 0 \,, \tag{S2}$$

where $[\widehat{A}, \widehat{B}]_{\pm} = \widehat{A}\widehat{B} \pm \widehat{B}\widehat{A}$, with the $(-)$ sign for bosons and the $(+)$ sign for fermions. These operators represent the quantized mode amplitudes of a quantum field. The operators

$$\widehat{n}_{\boldsymbol{s}} = \widehat{a}^{\dagger}_{\boldsymbol{s}} \widehat{a}_{\boldsymbol{s}}$$

are Hermitian number operators whose spectra consist of the natural numbers; they measure the number of particles occupying the mode labeled by $\boldsymbol{s}$. These operators act on a vector space spanned by Fock states of the form

$$|n_{\boldsymbol{s}_1}, n_{\boldsymbol{s}_2}, \ldots, n_{\boldsymbol{s}_i}, \ldots\rangle,$$

where the non-negative integer $n_{\boldsymbol{s}_i}$ denotes the number of particles in the single-particle state

$\boldsymbol{s}_i$. This space includes the *vacuum state*

$$|\mathbf{0}\rangle = |0, 0, 0, \ldots\rangle,$$

which contains no particles in any mode. The creation and annihilation operators introduced in Eq. (S2) act on these basis states according to

$$\widehat{a}_{\boldsymbol{s}_i}|n_{\boldsymbol{s}_1}, \ldots, n_{\boldsymbol{s}_i}, \ldots\rangle = \sqrt{n_{\boldsymbol{s}_i}}|n_{\boldsymbol{s}_1}, \ldots, n_{\boldsymbol{s}_i} - 1, \ldots\rangle, \tag{S3}$$

$$\widehat{a}^{\dagger}_{\boldsymbol{s}_i}|n_{\boldsymbol{s}_1}, \ldots, n_{\boldsymbol{s}_i}, \ldots\rangle = \sqrt{n_{\boldsymbol{s}_i} + 1}|n_{\boldsymbol{s}_1}, \ldots, n_{\boldsymbol{s}_i} + 1, \ldots\rangle. \tag{S4}$$

For fermionic creation and annihilation operators, the occupation numbers are restricted to $n_{\boldsymbol{s}} \in \{0, 1\}$, since the Pauli exclusion principle implies $(\widehat{a}^{\dagger}_{\boldsymbol{s}})^2 = 0$.

Representations of the Canonical (Anti)commutation Relations To define a quantum theory, one must specify a Hilbert space carrying a representation of the algebra of creation and annihilation operators. A crucial distinction arises between ordinary quantum mechanics, where a *finite* number of modes is involved, and quantum field theory (or many-body quantum mechanics) in the thermodynamic or continuum limit, where an *infinite* number of modes must be considered.

In the finite-mode case, which is typically encountered in practical quantum-chemical applications, the Fock space is constructed from occupation-number states of the form

$$|n_1, n_2, \ldots\rangle. \tag{S5}$$

Here the indices label a countable set of single-particle modes, such as atomic or molecular orbitals. The resulting Fock space is separable, and different choices of orthonormal single-particle bases correspond to different but unitarily equivalent representations of the same physical Hilbert space. This situation closely parallels the familiar freedom in quan-

tum chemistry to choose different orbital bases, which provide alternative but equivalent descriptions of the same many-electron state.

A qualitatively different situation arises when infinitely many degrees of freedom are involved. In this case, the canonical (anti)commutation relations admit *unitarily inequivalent representations*, which cannot be related to one another by unitary transformations acting within a single Hilbert space.

A general procedure for constructing a particular representation of the canonical (anti)commutation relations consists of:

1. Specifying an algebra of creation and annihilation operators $\{\hat{a}_s, \hat{a}_s^\dagger\}$ associated with an infinite set of modes.

2. Choosing a reference (vacuum) state $|\mathbf{0}\rangle$ such that

$$\hat{a}_s|\mathbf{0}\rangle = 0 \qquad \forall s\,. \tag{S6}$$

3. Constructing the vector space spanned by occupation-number states with finite particle number, for example

$$\mathcal{S} = \left\{ |n_1, n_2, \ldots\rangle \;\middle|\; \sum_i n_i = N,\ N \in \mathbb{N}_0 \right\}. \tag{S7}$$

The closure of the vector space generated by $\mathcal{S}$ defines a separable Hilbert space, usually referred to as the (bosonic or fermionic) Fock space associated with the chosen vacuum. In systems with infinitely many degrees of freedom, different choices of vacuum may lead to representations that are unitarily inequivalent. These inequivalent representations do not reflect different basis-set choices, but rather correspond to physically distinct realizations of the system, such as different vacuum states, broken-symmetry phases, or collective many-body backgrounds. A relevant example in this sense is provided by the Hilbert spaces of the electromagnetic field in the presence of different boundary conditions. This feature underlies

the emergence of quasiparticles, phase transitions, and effective field descriptions commonly encountered in condensed-matter physics and QED.[1,2,4]

Construction of Non-Relativistic QFTs Within this framework, the non-relativistic field operator $\widehat{\psi}(\boldsymbol{x},t)$ and its Hermitian conjugate $\widehat{\psi}^\dagger(\boldsymbol{x},t)$ are defined as

$$\widehat{\boldsymbol{\psi}}(\boldsymbol{x},t) = \sum_{\boldsymbol{s}} \boldsymbol{f}_{\boldsymbol{s}}(\boldsymbol{x})\, \widehat{a}_{\boldsymbol{s}}(t)\,, \tag{S8}$$

where $\boldsymbol{f}_{\boldsymbol{s}}(\boldsymbol{x}) = f_{\boldsymbol{s},i}\, \boldsymbol{e}_i$ are mode functions that transform in the same way under spatial rotations and spin transformations as the field operator $\widehat{\boldsymbol{\psi}}(\boldsymbol{x},t)$, i.e., they form a set of orthonormal spin orbitals. The field operators satisfy the equal-time (anti)commutation relations

$$[\widehat{\psi}_i(\boldsymbol{x},t), \widehat{\psi}_j^\dagger(\boldsymbol{y},t)]_\pm = \delta_{ij}\delta^{(3)}(\boldsymbol{x}-\boldsymbol{y}), \tag{S9}$$

$$[\widehat{\psi}_i(\boldsymbol{x},t), \widehat{\psi}_j(\boldsymbol{y},t)]_\pm = [\widehat{\psi}_i^\dagger(\boldsymbol{x},t), \widehat{\psi}_j^\dagger(\boldsymbol{y},t)]_\pm = 0\,. \tag{S10}$$

Here $[\cdot,\cdot]_-$ denotes commutators for bosons, and $[\cdot,\cdot]_+$ denotes anticommutators for fermions.

The mapping between the first-quantized, particle-based representation and the second-quantized, field-based representation can be constructed as follows for single-particle and two-particle operators:

$$\widehat{O}^{(I)}(\hat{\boldsymbol{p}},\hat{\boldsymbol{r}}) = \sum_{i,j} O^{(I)}_{ij}(\hat{\boldsymbol{p}},\hat{\boldsymbol{r}})\, \boldsymbol{e}_i \otimes \boldsymbol{e}_j, \tag{S11}$$

$$\widehat{O}^{(II)}(\hat{\boldsymbol{p}},\hat{\boldsymbol{r}};\hat{\boldsymbol{p}}',\hat{\boldsymbol{r}}') = \sum_{i,j,k,l} O^{(II)}_{ijkl}(\hat{\boldsymbol{p}},\hat{\boldsymbol{r}};\hat{\boldsymbol{p}}',\hat{\boldsymbol{r}}')\,(\boldsymbol{e}_i \otimes \boldsymbol{e}_j) \otimes (\boldsymbol{e}_k \otimes \boldsymbol{e}_l)\,, \tag{S12}$$

respectively. Expanding the field operators in an orthonormal single-particle basis $\{\boldsymbol{f}_{\boldsymbol{s}}\}$

yields

$$\hat{O}^{I} = \sum_{\boldsymbol{s},\boldsymbol{s}'} \langle \boldsymbol{f}_{\boldsymbol{s}} | \widehat{O}^{(I)}(\hat{\boldsymbol{r}}, \hat{\boldsymbol{p}}) | \boldsymbol{f}_{\boldsymbol{s}'} \rangle \, \widehat{a}^{\dagger}_{\boldsymbol{s}} \widehat{a}_{\boldsymbol{s}'} \tag{S13}$$

$$= \sum_{\boldsymbol{s},\boldsymbol{s}'} \left[\sum_{i,j} \int f^{*}_{\boldsymbol{s},i}(\boldsymbol{r}) \, O^{(I)}_{ij}(\boldsymbol{r}, -i\hbar\nabla_{\boldsymbol{r}}) \, f_{\boldsymbol{s}',j}(\boldsymbol{r}) \, \mathrm{d}^3\boldsymbol{r} \right] \widehat{a}^{\dagger}_{\boldsymbol{s}} \widehat{a}_{\boldsymbol{s}'},$$

$$\hat{O}^{II} = \frac{1}{2} \sum_{\boldsymbol{s},\boldsymbol{w},\boldsymbol{s}',\boldsymbol{w}'} \langle \boldsymbol{f}_{\boldsymbol{s}} \boldsymbol{f}_{\boldsymbol{w}} | \widehat{O}^{(II)}(\hat{\boldsymbol{p}}, \hat{\boldsymbol{r}}, \hat{\boldsymbol{p}}', \hat{\boldsymbol{r}}') | \boldsymbol{f}_{\boldsymbol{s}'} \boldsymbol{f}_{\boldsymbol{w}'} \rangle \widehat{a}^{\dagger}_{\boldsymbol{s}} \widehat{a}^{\dagger}_{\boldsymbol{w}} \widehat{a}_{\boldsymbol{s}'} \widehat{a}_{\boldsymbol{w}'} \tag{S14}$$

$$= \frac{1}{2} \sum_{\boldsymbol{s},\boldsymbol{w},\boldsymbol{s}',\boldsymbol{w}'} \left[\sum_{i,j,k,l} \iint \; f^{*}_{s,i}(\boldsymbol{r}) f^{*}_{w,j}(\boldsymbol{r}') O^{(II)}_{ijkl}(-i\hbar\nabla_{\boldsymbol{r}}, \boldsymbol{r}, -i\hbar\nabla_{\boldsymbol{r}'}, \boldsymbol{r}') f_{s',k}(\boldsymbol{r}) f_{w',l}(\boldsymbol{r}') \mathrm{d}^3\boldsymbol{r} \, \mathrm{d}^3\boldsymbol{r}' \right]$$

$$\times \, \widehat{a}^{\dagger}_{\boldsymbol{s}} \widehat{a}^{\dagger}_{\boldsymbol{w}} \widehat{a}_{\boldsymbol{s}'} \widehat{a}_{\boldsymbol{w}'}.$$

This mapping allows one to express any Hamiltonian operator $\hat{H}$ appearing in Schrödinger equations in a corresponding second-quantized form. Within this framework, the electronic Coulomb Hamiltonian generally adopted in quantum chemistry can be rewritten in the second-quantization formalism as

$$\widehat{H}_{\mathrm{Coul}} = \sum_{sw} h_{sw} \widehat{a}^{\dagger}_{s} \widehat{a}_{w} + \frac{1}{2} \sum_{ss'ww'} h_{sws'w'} \widehat{a}^{\dagger}_{s} \widehat{a}^{\dagger}_{w} \widehat{a}_{s'} \widehat{a}_{w'} \,, \tag{S15}$$

where the single-particle and two-particle Hamiltonian contributions read

$$h_{sw} = \int \psi^{*}_{s}(\boldsymbol{r}) \left(-\hbar^2 \frac{\nabla^2_{\boldsymbol{r}}}{2m_e} - \sum_{A=1}^{N_n} \frac{Z_A e^2}{4\pi\varepsilon_0 \|\boldsymbol{r} - \boldsymbol{R}_A\|} \right) \psi_w(\boldsymbol{r}) \, \mathrm{d}^3\boldsymbol{r}, \tag{S16}$$

$$h_{sws'w'} = e^2 \iint \frac{\psi^{*}_{s}(\boldsymbol{r}) \psi^{*}_{w}(\boldsymbol{r}') \psi_{s'}(\boldsymbol{r}) \psi_{w'}(\boldsymbol{r}')}{4\pi\varepsilon_0 \|\boldsymbol{r} - \boldsymbol{r}'\|} \, \mathrm{d}^3\boldsymbol{r} \, \mathrm{d}^3\boldsymbol{r}' \,. \tag{S17}$$

where $\{\psi_s(\boldsymbol{r})\}_s$ is a set of orthonormalized orbitals.

Construction of relativistic QFTs In relativistic field theories—such as quantum electrodynamics or the Dirac theory of electrons, which is essential in quantum chemistry for describing relativistic electrons—defining fields and Hamiltonian operators that transform

covariantly under Lorentz transformations involves significant subtleties. This helps explain the wide range of methods that have been proposed for constructing quantum field theory, including canonical quantization, path-integral quantization, and axiomatic quantum field theory.

A possible approach is to define the field operators for free theories, which transform according to finite-dimensional representations of the Lorentz group—e.g., scalar (spin-0), massive spin-1/2 (e.g., electrons), or massless spin-1 (e.g., photons)—as well as under discrete symmetry operations (parity, charge conjugation, and time reversal).

Local Hamiltonians consistent with special relativity can then be constructed by adding to the free Hamiltonian an interaction Hamiltonian density expressed as a local polynomial in the fields and their derivatives, while imposing the microcausality condition. Assuming that the Hamiltonian operator $\widehat{H}$ can be written in terms of a local Hamiltonian density $\widehat{h}(\boldsymbol{r}, t)$ such that

$$\widehat{H}(t) = \int_{\mathbb{R}^3} \widehat{h}(\boldsymbol{r}, t)\, \mathrm{d}^3\boldsymbol{r}, \tag{S18}$$

the Hamiltonian density operators evaluated at spacelike-separated spacetime points x and y must commute:

$$[\widehat{h}(x), \widehat{h}(y)] = 0 \quad \text{for} \quad (x-y)^2 < 0. \tag{S19}$$

Relativistic quantum field operators can be constructed starting from plane-wave solutions of relativistic wave equations (e.g., Klein–Gordon or Dirac), i.e.,

$$\widehat{\Phi}_\alpha(x) = \sum_\sigma \int \frac{\mathrm{d}^3\boldsymbol{p}}{(2\pi)^3\, 2E_{\boldsymbol{p}}} \left[u_\alpha(\boldsymbol{p}, \sigma)\, \widehat{a}(\boldsymbol{p}, \sigma)\, e^{-\frac{i}{\hbar} p\cdot x} + \overline{v}_\alpha(\boldsymbol{p}, \sigma)\, \widehat{b}^\dagger(\boldsymbol{p}, \sigma)\, e^{\frac{i}{\hbar} p\cdot x} \right], \tag{S20}$$

where

$$p^\mu = \left(\frac{E_{\boldsymbol{p}}}{c}, \boldsymbol{p} \right), \qquad E_{\boldsymbol{p}} = \sqrt{\boldsymbol{p}^2 c^2 + m^2 c^4}.$$

Here, α labels the components of a finite-dimensional Lorentz representation, σ labels spin/helicity states, and the functions $u_\alpha(\boldsymbol{p}, \sigma)$ and $v_\alpha(\boldsymbol{p}, \sigma)$ correspond to the components of

covariant amplitudes. Such amplitudes are built from irreducible finite-dimensional representations of the stabilizer (little) subgroup of the Lorentz group that leaves a given momentum p^μ invariant. This subgroup differs for massive and massless particles:

- For **massive particles**, the little group is isomorphic to $SO(3)$, the rotation group in the rest frame. States are labeled by mass $m > 0$ and spin $s = 0, \frac{1}{2}, 1, \ldots$, with $2s + 1$ spin states.

- For **massless particles**, the little group is isomorphic to the Euclidean group $ISO(2)$, and its finite-dimensional unitary representations are labeled by the *helicity* $\lambda = \pm s$ (for particles with definite spin s) and the momentum magnitude $|\boldsymbol{p}|$ (or, equivalently, the energy $E = |\boldsymbol{p}|c$). Helicity is the projection of spin along the direction of motion and is Lorentz-invariant for massless particles.

Note that, unlike in non-relativistic field theories, the relativistic field operator is a linear combination of creation and annihilation operators. This is a direct consequence of microcausality, which requires that field operators at spacelike separation commute (for bosons) or anticommute (for fermions), i.e.,

$$[\hat{\Phi}_\alpha(x), \hat{\Phi}^\dagger_\beta(y)]_\pm = 0 \qquad (x-y)^2 < 0\,. \tag{S21}$$

This structure also reflects the fact that each relativistic field generally describes two types of excitations: particles and antiparticles. The operator $\widehat{a}(\boldsymbol{p}, \sigma)$ annihilates a particle with momentum $\boldsymbol{p}$ and spin σ, while $\widehat{b}^\dagger(\boldsymbol{p}, \sigma)$ creates an antiparticle with the same quantum numbers. Their Hermitian conjugates, $\widehat{a}^\dagger(\boldsymbol{p}, \sigma)$ and $\widehat{b}(\boldsymbol{p}, \sigma)$, create particles and annihilate antiparticles, respectively. For neutral fields—i.e., fields that are their own antiparticles—the particle and antiparticle states coincide. In such cases, the field operator contains only one set of creation and annihilation operators. For example, Majorana fermions satisfy $\widehat{a}^{(\dagger)} = \widehat{b}^{(\dagger)}$, while the photon field (a real vector field) has no distinct antiparticle operator and is expanded solely in terms of $\widehat{a}$ and $\widehat{a}^\dagger$.

The (anti)commutation relations of the field operators follow from the canonical (anti)commutation relations imposed on the creation and annihilation operators, which for free fields read

$$[\widehat{a}(\boldsymbol{p},\sigma),\widehat{a}^{\dagger}(\boldsymbol{p}',\sigma')]_{\pm}=[\widehat{b}(\boldsymbol{p},\sigma),\widehat{b}^{\dagger}(\boldsymbol{p}',\sigma')]_{\pm}=(2\pi)^{3}\,2E_{\boldsymbol{p}}\,\delta^{(3)}(\boldsymbol{p}-\boldsymbol{p}')\,\delta_{\sigma\sigma'}\,, \tag{S22}$$

with all other (anti)commutators vanishing. The upper (lower) sign refers to fermionic (bosonic) fields, in accordance with the spin–statistics theorem.

Interacting fields and physical quasi-particle fields Finally, it is worth emphasizing that in quantum field theory there exists a *weak operator identity*, denoted by $\stackrel{W}{=}$, which holds only at the level of matrix elements between states of a complete basis. This weak identity relates two sets of field operators acting on Fock space: the interacting Heisenberg fields $\{\widehat{\psi}_{A}\}_{A=1,\dots,N}$ and the physical quasi-particle fields $\{\widehat{\phi}_{a}\}_{a=1,\dots,N}$. Explicitly, one may write

$$\begin{aligned}\widehat{\psi}_{A}(x)\stackrel{W}{=}\rho_{A}(x)\,\widehat{\mathbb{I}}+\sum_{a}Z_{Aa}^{1/2}\,\widehat{\phi}_{a}(x)+\sum_{ab}\int \mathrm{d}^{4}y_{1}\,\mathrm{d}^{4}y_{2}\,K_{Aab}(x;y_{1},y_{2})\,:\widehat{\phi}_{a}(y_{1})\widehat{\phi}_{b}(y_{2}):\\ +\sum_{abc}\int \mathrm{d}^{4}y_{1}\,\mathrm{d}^{4}y_{2}\,\mathrm{d}^{4}y_{3}\,K_{Aabc}(x;y_{1},y_{2},y_{3})\,:\widehat{\phi}_{a}(y_{1})\widehat{\phi}_{b}(y_{2})\widehat{\phi}_{c}(y_{3}):+\cdots\,.\end{aligned} \tag{S23}$$

Here $\rho_{A}(x)$ is a c-number function, which can be non-vanishing only for bosonic fields, $Z_{Aa}^{1/2}$ are field-strength renormalization constants, and $K_{Aab}(x;y_{1},y_{2})$, $K_{Aabc}(x;y_{1},y_{2},y_{3})$, ... are (generally nonlocal) convolution kernels. Normal ordering is defined with respect to the vacuum of the asymptotic quasi-particle fields. The kernels may be chosen such that, when evaluated between physical quasi-particle states, the interacting Hamiltonian is weakly equivalent to an effective free Hamiltonian expressed in terms of the creation and annihilation operators $\hat{a}_{s}^{\dagger},\hat{a}_{s}$ associated with the quasi-particle fields, namely

$$\widehat{H}[\{\widehat{\psi}_{A}\}_{A}]\stackrel{W}{=}\sum_{s}h_{s}\left(\hat{a}_{s}^{\dagger}\hat{a}_{s}\pm\frac{1}{2}\right)=\widehat{H}_{0}[\{\widehat{\phi}_{a}\}_{a}]\,, \tag{S24}$$

where the plus sign applies to bosonic modes and the minus sign to fermionic modes. In quantum chemistry and condensed-matter applications, this weak equivalence underlies the practical success of quasiparticle descriptions, in which interacting many-electron systems are effectively described in terms of dressed single-particle excitations, despite the absence of exact integrability. Such a conceptual picture provides a formal justification for the widespread use of effective single-particle descriptions, normal ordering with respect to a reference determinant, and perturbative or coupled-cluster expansions in electronic-structure theory. In Green's-function formulations, the same structure appears through the emergence of quasiparticle poles of the one-body propagator, corresponding to dressed electronic excitations with renormalized energies and spectral weights.

For applications in quantum chemistry, the relevant relativistic fields are the fermionic relativistic field—representing relativistic electrons, particularly core electrons in heavy elements—and the electromagnetic field.

S2 Dirac Free Equation

The free field describing relativistic fermions is the Dirac field $\widehat{\psi}(x)$, a four-component complex spinor field satisfying the Dirac equation

$$i\hbar\,\gamma^\mu \partial_\mu \psi(x) - mc\,\psi(x) = 0\,, \tag{S25}$$

where $x^0 = ct$ is the time coordinate, x^i $(i = 1, 2, 3)$ are spatial coordinates, and γ^μ are the 4×4 Dirac matrices in the Dirac representation, satisfying the Clifford algebra relation $\{\gamma^\mu, \gamma^\nu\} = 2\eta^{\mu\nu}\mathbb{I}_{4\times 4}$, i.e.,

$$\gamma^0 = \begin{pmatrix} \mathbb{I}_2 & 0 \\ 0 & -\mathbb{I}_2 \end{pmatrix}, \qquad \gamma^i = \begin{pmatrix} 0 & \sigma_i \\ -\sigma_i & 0 \end{pmatrix}, \tag{S26}$$

with $\mathbb{I}_2$ the 2×2 identity matrix and σ_i the Pauli matrices

$$\sigma_1 = \begin{pmatrix} 0 & 1 \\ 1 & 0 \end{pmatrix}, \quad \sigma_2 = \begin{pmatrix} 0 & -i \\ i & 0 \end{pmatrix}, \quad \sigma_3 = \begin{pmatrix} 1 & 0 \\ 0 & -1 \end{pmatrix}. \tag{S27}$$

The Dirac field admits a mode decomposition of the form in Eq. (S20), where $u = u(p)$ and $v = v(p)$ are four-component spinors satisfying

$$(\gamma^\mu p_\mu - mc\,\mathbb{I}_4)\,\boldsymbol{u}(p) = 0, \qquad (\gamma^\mu p_\mu + mc\,\mathbb{I}_4)\,\boldsymbol{v}(p) = 0\,. \tag{S28}$$

Applying minimal coupling to the Dirac Hamiltonian yields the charge density and current density operators

$$\widehat{\rho}(\boldsymbol{r}, t) = -e\,\widehat{\overline{\psi}}(\boldsymbol{r}, t)\gamma^0\widehat{\psi}(\boldsymbol{r}, t), \tag{S29}$$

$$\widehat{\boldsymbol{J}}(\boldsymbol{r}, t) = -e\,c\,\widehat{\overline{\psi}}(\boldsymbol{r}, t)\,\boldsymbol{\gamma}\,\widehat{\psi}(\boldsymbol{r}, t), \tag{S30}$$

where $\widehat{\overline{\psi}} = \widehat{\psi}^\dagger\gamma^0$ and $\boldsymbol{\gamma} = (\gamma^1, \gamma^2, \gamma^3)$. In many quantum-chemical applications, one works in a no-pair approximation, so that the field is projected onto the positive-energy (electron) subspace and the charge and current densities can be expressed solely in terms of electron creation and annihilation operators $\widehat{a}(\boldsymbol{p}, s)$ and $\widehat{a}^\dagger(\boldsymbol{p}, s)$, with reference to Eq. (S20). Relativistic electrons in the presence of an external electric field generated by atomic nuclei (or a more general electromagnetic field, if nuclear total angular momentum is included) are usually described by effective Hamiltonians derived from a full relativistic quantum electrodynamics picture. The mathematical treatment required to derive the spectrum of these theories goes beyond the scope of the current review, and we refer to the specialized literature for further details.[5–7]

S3 Molecular Quantum Electrodynamics in a Nutshell

The multipolar, or Power–Zienau–Woolley (PZW), formalism for nonrelativistic electrodynamics is obtained through a canonical transformation of the classical electromagnetic–matter phase space. Upon quantization, this transformation is implemented by a unitary operator. The resulting representation is particularly well suited to describing the interaction between localized, distinguishable charge distributions and the electromagnetic field in terms of electric and magnetic multipole moments.

The central idea is to formulate the coupling between matter and the electromagnetic field (EMF) through the polarization $\boldsymbol{P}(\boldsymbol{r}, t)$ and magnetization $\boldsymbol{M}(\boldsymbol{r}, t)$ fields, which are composite quantities constructed from the microscopic degrees of freedom of matter, in close analogy with macroscopic molecular electrodynamics. From a mathematical perspective, this construction is naturally embedded in the four-dimensional covariant formulation of Maxwell's equations in terms of the Faraday tensor

$$F_{\mu\nu} = \partial_\mu A_\nu - \partial_\nu A_\mu \, , \tag{S31}$$

where $\mu, \nu = 0, 1, 2, 3$ label the Cartesian spacetime coordinates (ct, x, y, z), and indices are raised and lowered using the Minkowski metric $\eta_{\mu\nu} = \mathrm{diag}(1, -1, -1, -1)$. The four-current is defined as $J^\mu = (c\rho, \boldsymbol{J})$, and Maxwell's equations can be written compactly as

$$\partial_\mu F^{\mu\nu} = \mu_0 J^\nu, \qquad \partial_{[\mu} F_{\nu\rho]} = 0 \, , \tag{S32}$$

where square brackets on the indices indicate full antisymmetrization.

Within this framework, it is natural to introduce an antisymmetric rank-two polarization tensor $P^{\mu\nu}$ such that

$$J^\nu \equiv J^\nu_{\mathrm{free}} + \partial_\mu P^{\mu\nu} \, , \tag{S33}$$

with the supplementary requirement that the continuity equation be satisfied,

$$\partial_\mu J^\mu = \partial_\mu J^\mu_{\mathrm{free}} + \partial_\mu \partial_\nu P^{\mu\nu} = 0\,. \tag{S34}$$

Assuming that all charges are bound, so that there are no freely moving charges or currents, the charge and current associated with freely moving charges vanish, i.e., $J^\mu_{\mathrm{free}} = 0$. In this case, the antisymmetric tensor

$$P^{0i} = P^i, \qquad P^{ij} = \epsilon^{ijk} M_k, \qquad P^{\mu\nu} = -P^{\nu\mu}, \tag{S35}$$

leads directly to the constitutive relations for the polarization and magnetization fields,

$$\nabla \cdot \boldsymbol{P}(\boldsymbol{r},t) = -\rho(\boldsymbol{r},t), \tag{S36}$$

$$\nabla \times \boldsymbol{M}(\boldsymbol{r},t) = \boldsymbol{J}(\boldsymbol{r},t) - \partial_t \boldsymbol{P}(\boldsymbol{r},t). \tag{S37}$$

Equations (S36) and (S37) do not uniquely define the polarization (and, consequently, the magnetization) field. For bounded charge distributions, the polarization depends on the arbitrary choice of a reference point (pole of reduction). For a system of N_{mol} molecules, we introduce one pole $\boldsymbol{O}_\xi$ for each molecule $\xi = 1, \ldots, N_{\mathrm{mol}}$ and write

$$\boldsymbol{P}(\boldsymbol{r},t) = \sum_{\xi=1}^{N_{\mathrm{mol}}} \boldsymbol{P}_\xi(\boldsymbol{r},t;\boldsymbol{O}_\xi), \qquad \boldsymbol{M}(\boldsymbol{r},t) = \sum_{\xi=1}^{N_{\mathrm{mol}}} \boldsymbol{M}_\xi(\boldsymbol{r},t;\boldsymbol{O}_\xi), \tag{S38}$$

together with $\rho(\boldsymbol{r},t) = \sum_\xi \rho_\xi(\boldsymbol{r},t)$ and $\boldsymbol{J}(\boldsymbol{r},t) = \sum_\xi \boldsymbol{J}_\xi(\boldsymbol{r},t)$.

In general,

$$\boldsymbol{P}_\xi(\boldsymbol{r},t;\boldsymbol{O}_\xi) = \int \boldsymbol{\mathcal{G}}(\boldsymbol{r},\boldsymbol{r}';\boldsymbol{O}_\xi)\,\rho_\xi(\boldsymbol{r}',t)\,\mathrm{d}^3\boldsymbol{r}'\,, \tag{S39}$$

where $\boldsymbol{\mathcal{G}}(\boldsymbol{r},\boldsymbol{r}';\boldsymbol{O}_\xi)$ is a vector-valued Green function satisfying

$$\nabla_{\boldsymbol{r}} \cdot \boldsymbol{\mathcal{G}}(\boldsymbol{r},\boldsymbol{r}';\boldsymbol{O}_\xi) = -\delta^{(3)}(\boldsymbol{r}-\boldsymbol{r}')\,.$$

An explicit representation is

$$\boldsymbol{\mathcal{G}}(\boldsymbol{r},\boldsymbol{r}';\boldsymbol{O}_\xi) = \boldsymbol{\mathcal{G}}^{\parallel}(\boldsymbol{r},\boldsymbol{r}';\boldsymbol{O}_\xi) + \int_0^1 (\boldsymbol{r}'-\boldsymbol{O}_\xi)\,\delta^{(3)}\big[(\boldsymbol{O}_\xi-\boldsymbol{r})+\lambda(\boldsymbol{r}'-\boldsymbol{O}_\xi)\big]\,\mathrm{d}\lambda\,, \tag{S40}$$

where

$$\boldsymbol{\mathcal{G}}^{\parallel}(\boldsymbol{r},\boldsymbol{r}';\boldsymbol{O}_\xi) = -\nabla_{\boldsymbol{r}}\left[\frac{1}{4\pi\|\boldsymbol{r}-\boldsymbol{r}'\|}\right]$$

denotes the longitudinal component of the Green function, while the integral term represents its transverse contribution.

With these conventions, the polarization field may be written as

$$\boldsymbol{P}_\xi(\boldsymbol{r},t;\boldsymbol{O}_\xi) = Q_\xi\,\boldsymbol{\mathcal{G}}^{\parallel}(\boldsymbol{r};\boldsymbol{O}_\xi) \tag{S41}$$

$$+\iint_0^1 (\boldsymbol{r}'-\boldsymbol{O}_\xi)\,\delta^{(3)}\big[\lambda(\boldsymbol{r}'-\boldsymbol{O}_\xi)-(\boldsymbol{r}-\boldsymbol{O}_\xi)\big]\,\rho_\xi(\boldsymbol{r}',t)\,\mathrm{d}\lambda\,\mathrm{d}^3\boldsymbol{r}'\,, \tag{S42}$$

where $Q_\xi = \int \rho_\xi(\boldsymbol{r},t)\,\mathrm{d}^3\boldsymbol{r}$ is the total charge of molecule ξ. This formalism is particularly relevant for electrically neutral molecules ($Q_\xi = 0$).

From the above definitions and Eq. (S37), it follows that the magnetization field can be written in SI units as

$$\boldsymbol{M}_\xi(\boldsymbol{r},t) = \int \frac{\mu_0}{4\pi}\,\frac{\nabla_{\boldsymbol{r}'}\times\boldsymbol{J}_\xi(\boldsymbol{r}',t)-\nabla_{\boldsymbol{r}'}\times\boldsymbol{K}_\xi(\boldsymbol{r}',t)}{\|\boldsymbol{r}-\boldsymbol{r}'\|}\,\mathrm{d}^3\boldsymbol{r}'\,, \tag{S43}$$

where

$$\boldsymbol{K}_\xi(\boldsymbol{r},t) = \int \boldsymbol{\mathcal{G}}(\boldsymbol{r},\boldsymbol{r}';\boldsymbol{O}_\xi)\,\partial_t\rho_\xi(\boldsymbol{r}',t)\,\mathrm{d}^3\boldsymbol{r}'\,. \tag{S44}$$

In general, the polarization and magnetization fields are origin dependent. This could raise concerns regarding the applicability of such a representation to the characterization of charge and current density distributions in matter. However, this origin dependence does not affect physical couplings.

For instance, for an external electrostatic potential $\phi(\boldsymbol{r})$, one has

$$\int \rho_\xi(\boldsymbol{r})\,\phi(\boldsymbol{r})\,\mathrm{d}^3\boldsymbol{r} = -\int \left[\nabla\cdot\boldsymbol{P}_\xi(\boldsymbol{r};\boldsymbol{O}_\xi)\right]\phi(\boldsymbol{r})\,\mathrm{d}^3\boldsymbol{r} = -\int \boldsymbol{P}_\xi(\boldsymbol{r};\boldsymbol{O}_\xi)\cdot\boldsymbol{E}(\boldsymbol{r})\,\mathrm{d}^3\boldsymbol{r}, \tag{S45}$$

where $\boldsymbol{E}(\boldsymbol{r}) = -\nabla\phi(\boldsymbol{r})$, and surface terms are assumed to vanish (for localized charge distributions or appropriate boundary conditions). The left-hand side is manifestly independent of the choice of $\boldsymbol{O}_\xi$; therefore, the contracted form of the interaction is origin independent. Analogous considerations apply to magnetostatic and fully electromagnetic couplings.

We now show how the nonrelativistic QED Hamiltonian in the Coulomb gauge, formulated in the minimal-coupling representation, can be mapped to an equivalent Hamiltonian formulation in the multipolar coupling.

We begin with the minimal-coupling Lagrangian for a system of N_{mol} molecules labelled by ξ, each associated with a pole of reduction $\boldsymbol{O}_\xi$. Molecule ξ consists of N_ξ nonrelativistic charged particles labelled by $A_\xi = 1,\ldots,N_\xi$, interacting with the electromagnetic field,

$$\begin{aligned}\mathcal{L}_{\min} = &\sum_{\xi=1}^{N_{\mathrm{mol}}}\sum_{A_\xi=1}^{N_\xi}\left[\frac{1}{2}m_{A_\xi}\dot{\boldsymbol{r}}_{A_\xi}^2 + q_{A_\xi}\dot{\boldsymbol{r}}_{A_\xi}\cdot\boldsymbol{A}(\boldsymbol{r}_{A_\xi},t) - q_{A_\xi}\phi(\boldsymbol{r}_{A_\xi},t)\right]\\ &+\frac{\varepsilon_0}{2}\int\left[\|\boldsymbol{E}(\boldsymbol{r},t)\|^2 - c^2\|\boldsymbol{B}(\boldsymbol{r},t)\|^2\right]\mathrm{d}^3\boldsymbol{r}\,.\end{aligned} \tag{S46}$$

Here $\phi(\boldsymbol{r},t)$ and $\boldsymbol{A}(\boldsymbol{r},t)$ are the scalar and vector potentials. In the Coulomb gauge, $\nabla\cdot\boldsymbol{A}(\boldsymbol{r},t) = 0$, the scalar potential satisfies Poisson's equation $\nabla^2\phi(\boldsymbol{r},t) = -\rho(\boldsymbol{r},t)/\varepsilon_0$, and the electric and magnetic fields decompose as

$$\begin{aligned}E_i(\boldsymbol{r},t) &= \sum_{j=1}^{3}\int\left[\delta_{ij}^{\perp}(\boldsymbol{r}-\boldsymbol{r}') + (\delta_{ij} - \delta_{ij}^{\perp}(\boldsymbol{r}-\boldsymbol{r}'))\right]E_j(\boldsymbol{r}',t)\,\mathrm{d}^3\boldsymbol{r}'\\ &= E_{\perp,i}(\boldsymbol{r},t) + E_{\|,i}(\boldsymbol{r},t),\\ \boldsymbol{E}_{\|}(\boldsymbol{r},t) &= -\nabla\phi(\boldsymbol{r},t), \qquad \boldsymbol{E}_{\perp}(\boldsymbol{r},t) = -\partial_t\boldsymbol{A}(\boldsymbol{r},t),\\ \boldsymbol{B}(\boldsymbol{r},t) &= \nabla\times\boldsymbol{A}(\boldsymbol{r},t)\,.\end{aligned}$$

Using the decomposition of the current in terms of polarization and magnetization fields, the same equations of motion can be obtained from the multipolar (PZW) Lagrangian

$$\begin{aligned}\mathcal{L}_{\mathrm{PZW}} = \sum_{\xi=1}^{N_{\mathrm{mol}}} \left[\sum_{A_\xi=1}^{N_\xi} \frac{1}{2} m_{A_\xi} \dot{\boldsymbol{r}}_{A_\xi}^2 + \sum_{B_\xi < A_\xi} V_{\mathrm{Coul}}(\boldsymbol{r}_{A_\xi}, \boldsymbol{r}_{B_\xi}) + \sum_{\xi' < \xi} \sum_{B_{\xi'}=1}^{N_{\xi'}} V_{\mathrm{Coul}}(\boldsymbol{r}_{A_\xi}, \boldsymbol{r}_{B_{\xi'}}) \right] \\ + \frac{\varepsilon_0}{2} \int \left(\|\boldsymbol{E}(\boldsymbol{r},t)\|^2 - c^2 \|\boldsymbol{B}(\boldsymbol{r},t)\|^2 \right) \mathrm{d}^3\boldsymbol{r} + \int \left[\boldsymbol{P}(\boldsymbol{r},t) \cdot \boldsymbol{E}(\boldsymbol{r},t) + \boldsymbol{M}(\boldsymbol{r},t) \cdot \boldsymbol{B}(\boldsymbol{r},t) \right] \mathrm{d}^3\boldsymbol{r}\,. \end{aligned} \tag{S47}$$

The two Lagrangians (S46) and (S47) are related by a total time derivative (the Göppert–Mayer term),

$$\mathcal{L}_{\mathrm{PZW}} = \mathcal{L}_{\mathrm{min}} + \frac{\mathrm{d}}{\mathrm{d}t} \sum_{\xi=1}^{N_{\mathrm{mol}}} \int \boldsymbol{P}_\xi(\boldsymbol{r}, t; \boldsymbol{O}_\xi) \cdot \boldsymbol{A}(\boldsymbol{r}, t)\, \mathrm{d}^3\boldsymbol{r}\,. \tag{S48}$$

Passing to the Hamiltonian formulation requires defining the conjugate momenta of the matter and field degrees of freedom, which read, respectively,

$$p_{A_\xi,i} = \frac{\partial \mathcal{L}_{\mathrm{min}}}{\partial \dot{r}_{A_\xi,i}} = m_{A_\xi} \dot{r}_{A_\xi,i} + q_{A_\xi} A_i(\boldsymbol{r}_{A_\xi}, t), \tag{S49}$$

$$\Pi_i(\boldsymbol{r}) = \frac{\partial \mathcal{L}_{\mathrm{min}}}{\partial \dot{A}_i(\boldsymbol{r})} = \varepsilon_0 \dot{A}_i(\boldsymbol{r}) = -\varepsilon_0 E_{\perp,i}(\boldsymbol{r})\,. \tag{S50}$$

Applying the Legendre transform to the minimal-coupling Lagrangian, the minimal-coupling Hamiltonian reads

$$\begin{aligned} H_{\mathrm{min}} = \sum_{\xi=1}^{N_{\mathrm{mol}}} \sum_{A_\xi=1}^{N_\xi} \frac{1}{2m_{A_\xi}} \left\| \boldsymbol{p}_{A_\xi} - q_{A_\xi} \boldsymbol{A}(\boldsymbol{r}_{A_\xi}) \right\|^2 + \sum_{\xi=1}^{N_{\mathrm{mol}}} \sum_{A_\xi=1}^{N_\xi} q_{A_\xi} \phi(\boldsymbol{r}_{A_\xi}) \\ + \frac{1}{2} \int \left(\frac{\|\boldsymbol{\Pi}(\boldsymbol{r})\|^2}{\varepsilon_0} + \varepsilon_0 c^2 \|\boldsymbol{B}(\boldsymbol{r})\|^2 \right) \mathrm{d}^3\boldsymbol{r}\,. \end{aligned} \tag{S51}$$

The canonical conjugate momenta derived from the Lagrangian in the multipolar coupling

read

$$p_{A_\xi,i} = \frac{\partial \mathcal{L}_{\mathrm{PZW}}}{\partial \dot{r}_{A_\xi,i}} = m_{A_\xi} \dot{r}_{A_\xi,i} - \sum_{j,k} \int \epsilon_{ijk}\, n_{A_\xi,j}(\boldsymbol{r})\, B_k(\boldsymbol{r})\, \mathrm{d}^3\boldsymbol{r}, \tag{S52}$$

$$\Pi_i(\boldsymbol{r}) = \frac{\partial \mathcal{L}_{\mathrm{PZW}}}{\partial \dot{A}_i(\boldsymbol{r})} = \varepsilon_0 \dot{A}_i(\boldsymbol{r}) - \boldsymbol{P}_i^{\perp}(\boldsymbol{r}) = -\boldsymbol{D}_i^{\perp}(\boldsymbol{r})\,, \tag{S53}$$

where ϵ_{ijk} is the Levi-Civita symbol and $\boldsymbol{D}(\boldsymbol{r})$ is the displacement field. Here $\boldsymbol{n}_{A_\xi}$ is a vector field of the form

$$\boldsymbol{n}_{A_\xi}(\boldsymbol{r}) = -q_{A_\xi}(\boldsymbol{r} - \boldsymbol{O}_\xi) \int_0^1 \lambda\, \delta\big(\boldsymbol{r} - \boldsymbol{O}_\xi - \lambda(\boldsymbol{r}_{A_\xi} - \boldsymbol{O}_\xi)\big)\, \mathrm{d}\lambda\,. \tag{S54}$$

By performing the Legendre transform of the Lagrangian $\mathcal{L}_{\mathrm{PZW}}$, the multipolar Hamiltonian is given by

$$\begin{aligned} H_{\mathrm{PZW}} = &\sum_{\xi=1}^{N_{\mathrm{mol}}} \sum_{A_\xi=1}^{N_\xi} \frac{\|\boldsymbol{p}_{A_\xi}\|^2}{2m_{A_\xi}} + \frac{1}{2\varepsilon_0} \int \left\|\boldsymbol{\Pi}(\boldsymbol{r}) + \boldsymbol{P}^{\perp}(\boldsymbol{r})\right\|^2 \mathrm{d}^3\boldsymbol{r} \\ &+ \frac{1}{2\mu_0} \int \|\boldsymbol{B}(\boldsymbol{r})\|^2\, \mathrm{d}^3\boldsymbol{r} - \int \boldsymbol{M}(\boldsymbol{r},t) \cdot \boldsymbol{B}(\boldsymbol{r},t)\, \mathrm{d}^3\boldsymbol{r} \\ &+ \frac{1}{2} \sum_{i,j} \iint B_i(\boldsymbol{r})\, O_{ij}(\boldsymbol{r},\boldsymbol{r}')\, B_j(\boldsymbol{r}')\, \mathrm{d}^3\boldsymbol{r}\, \mathrm{d}^3\boldsymbol{r}'\,, \end{aligned} \tag{S55}$$

where $O_{ij}(\boldsymbol{r},\boldsymbol{r}')$ is the diamagnetic interaction kernel,

$$O_{ij}(\boldsymbol{r},\boldsymbol{r}') = \sum_{\xi=1}^{N_{\mathrm{mol}}} \sum_{A_\xi=1}^{N_\xi} \frac{1}{m_{A_\xi}} \sum_{k,l,m} \epsilon_{ikl}\epsilon_{jml}\, n_{A_\xi,k}(\boldsymbol{r})\, n_{A_\xi,m}(\boldsymbol{r}')\,. \tag{S56}$$

It is important to note that the PZW Hamiltonian contains a self-energy term for the polarization field, $\frac{1}{2\varepsilon_0}\int \|\boldsymbol{P}^{\perp}(\boldsymbol{r},t)\|^2\, \mathrm{d}^3\boldsymbol{r}$, which is particularly important because it contributes to making the Hamiltonian bounded from below. Moreover, if the overlap of charge densities can be neglected, it can be shown that the transverse and longitudinal components of the direct interaction between polarization fields of two different molecules mutually cancel, i.e.,

$\int \boldsymbol{P}^{\perp}_{\xi}(\boldsymbol{r}) \cdot \boldsymbol{P}^{\perp}_{\xi'}(\boldsymbol{r}) = -\int \boldsymbol{P}^{\parallel}_{\xi}(\boldsymbol{r}) \cdot \boldsymbol{P}^{\parallel}_{\xi'}(\boldsymbol{r})$. It follows that the matter fields representing the molecular charge distributions are coupled only to the electromagnetic field. For this reason, the multipolar coupling framework is particularly suited to investigating intermolecular interactions and, more generally, the QED of bounded systems of charges.

The two Hamiltonians are related by a canonical transformation which, in the quantum theory, is implemented by the unitary operator

$$\hat{U}_{\mathrm{GM}}(t) = \exp\left[\frac{i}{\hbar} \int \hat{\boldsymbol{P}}(\boldsymbol{r}, t) \cdot \hat{\boldsymbol{A}}(\boldsymbol{r}, t) \, \mathrm{d}^3\boldsymbol{r}\right] . \tag{S57}$$

Here the two operators are evaluated at a fixed time t.

It should be noted that the length gauge, widely used in polaritonic chemistry and cavity quantum electrodynamics, corresponds to the multipolar representation truncated at electric-dipole order in the long-wavelength approximation.

Accordingly, operators transform as

$$\hat{O}_{\mathrm{PZW}} = \hat{U}_{\mathrm{GM}} \, \hat{O}_{\min} \, \hat{U}^{\dagger}_{\mathrm{GM}},$$

and, in particular, the canonical field momentum transforms as

$$\hat{\boldsymbol{\Pi}}^{(\mathrm{PZW})}(\boldsymbol{r}) = \hat{U}_{\mathrm{GM}} \, \hat{\boldsymbol{\Pi}}^{(\min)}(\boldsymbol{r}) \, \hat{U}^{\dagger}_{\mathrm{GM}} = \hat{\boldsymbol{\Pi}}^{(\min)}(\boldsymbol{r}) + \hat{\boldsymbol{P}}^{\perp}(\boldsymbol{r}) \, .$$

The transverse canonical commutation relations of the electromagnetic field

$$\left[\hat{A}_i(\boldsymbol{r}), \hat{\Pi}_j(\boldsymbol{r}')\right] = i\hbar \, \delta^{\perp}_{ij}(\boldsymbol{r} - \boldsymbol{r}') \, , \tag{S58}$$

where $\delta^{\perp}_{ij}(\boldsymbol{r} - \boldsymbol{r}')$ is the dyadic projector operator, are preserved under the transformation, since electromagnetic-field operators commute with matter operators. For further details on multipolar coupling, we refer the reader to the literature.[8–10]

References

(1) Umezawa, H.; Matsumoto, H.; Tachiki, M. *Thermo Field Dynamics and Condensed States*; North-Holland Publishing Co., 1982.

(2) Blasone, M.; Jizba, P.; Vitiello, G. *Quantum Field Theory and Its Macroscopic Manifestations*; Imperial College Press, 2011.

(3) Duncan, A. *The Conceptual Framework of Quantum Field Theory*; Oxford University Press, 2012.

(4) Umezawa, H. *Advanced Field Theory: Micro, Macro, and Thermal Physics*; AIP Press, 1995.

(5) Roos, B. O.; Malmqvist, P.-Å. Relativistic quantum chemistry: the multiconfigurational approach. *Physical Chemistry Chemical Physics* **2004**, *6*, 2919–2927.

(6) Reiher, M.; Wolf, A. *Relativistic Quantum Chemistry: The Fundamental Theory of Molecular Science*; Wiley, 2015.

(7) Liu, W. Essentials of relativistic quantum chemistry. *The Journal of Chemical Physics* **2020**, *152*, 180901.

(8) Craig, D. P.; Thirunamachandran, T. *Molecular Quantum Electrodynamics: An Introduction to Radiation-Molecule Interactions*; Academic Press, 1984.

(9) Salam, A. *Molecular Quantum Electrodynamics*; Wiley, 2010.

(10) Woolley, R. G. *Foundations of Molecular Quantum Electrodynamics*; Cambridge University Press, 2022.
